# A redox-active ionic liquid manifesting charge-transfer interaction between a viologen and carbazole and its effect on the viscosity, ionic conductivity, and redox process of the viologen†

**DOI:** 10.1039/d0sc06244h

**Published:** 2021-02-18

**Authors:** Hironobu Tahara, Yudai Tanaka, Shoko Yamamoto, Shigeki Yonemori, Bun Chan, Hiroto Murakami, Takamasa Sagara

**Affiliations:** Graduate School of Engineering, Nagasaki University 1-14 Bunkyo Nagasaki 852-8521 Japan h-tahara@nagasaki-u.ac.jp sagara@nagasaki-u.ac.jp; School of Engineering, Nagasaki University 1-14 Bunkyo Nagasaki 852-8521 Japan

## Abstract

Redox-active ionic liquids (RAILs) are gaining attention as a material that can create a wide range of functions. We herein propose a charge-transfer (CT) RAIL by mixing two RAILs, specifically a carbazole-based ionic liquid ([CzC_4_ImC_1_][TFSI]) as a donor and a viologen-based ionic liquid ([C_4_VC_7_][TFSI]_2_) as an acceptor. We investigated the effect of CT interaction on the physicochemical properties of the CT ionic liquid (CT-IL) using the results of temperature-dependent measurements of UV-vis absorption, viscosity, and ionic conductivity as well as cyclic voltammograms. We employed the Walden analysis and the Grunberg–Nissan model to elucidate the effect of the CT interaction on the viscosity and ionic conductivity. The CT interaction reduces the viscosity by reducing the electrostatic attraction between the dicationic viologen and TFSI anion. It also reduces the ionic conductivity by the CT association of the dicationic viologen and carbazole. The electrochemically reversible responses of the viologens in [C_4_VC_7_][TFSI]_2_ and CT-IL are consistent with the Nernstian and the interacting two-redox site models. Notably, the transport and electrochemical properties are modulated by CT interaction, leading to unique features that are not present in individual component ILs. The inclusion of CT interaction in RAILs thus provides a powerful means to expand the scope of functionalized ionic liquids.

## Abstract 2

A redox-active ionic liquid (RAIL) consisting of a carbazole and viologen shows charge transfer (CT) interaction. The physicochemical properties are modulated by the CT interaction by comparison with the individual RAILs.

## Graphical abstract

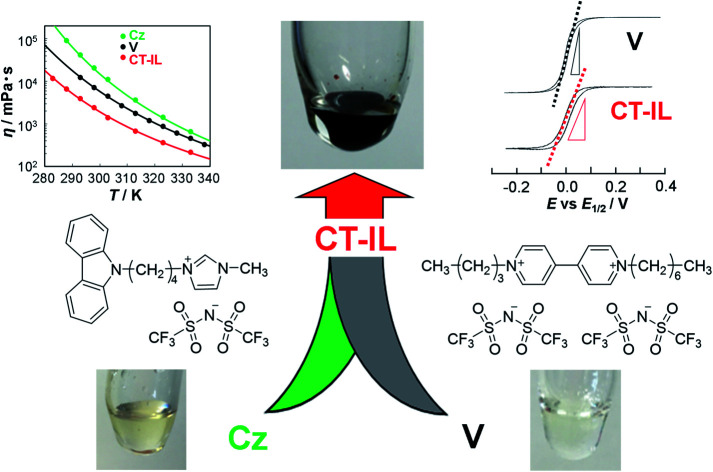

## 1. Introduction

Charge transfer (CT) interaction between a donor and acceptor has been of great interest because it can determine the material structures, electric conduction, and photoconduction in solid state.^1^ In addition, it can give rise to unique dynamical physicochemical properties of Stoddart's supramolecules,^2,3^ intermediate complex of chemical reaction,^4^ and photochemically active species^5–8^ in solution phase. CT interaction never emerges in its individual component species but appears as a synergetic effect. The CT interaction depends on the combination of a donor and acceptor, and one can exploit such combinations in ionic liquids to produce new photoactivity and to modulate their physicochemical properties.

A myriad of ionic liquids (ILs) with various physicochemical properties have been synthesized. Because ILs are designer solvents^9^ that can be readily created by combining organic cations and anions, one can straightforwardly tune their viscosity, ionic conductivity, and melting point, as well as the solubility of substrate molecules. This leads to some notable advantages over typical organic solvents. For example, ILs are often preferred due to the higher solubility of solutes such as cellulose^10^ and CO_2_.^11,12^ Therefore, they are frequently applied to extraction and separation chemistry.^13^ ILs also provide interesting functionalities rarely found in organic molecular solvents,^14^ leading to unique IL-based liquid materials. In addition, organic cations can be modified to further transform ILs into functionalized ionic liquids (FILs) or task-specific ionic liquids.^15^

Extensive developments of FILs have led to functional liquid materials that are not merely a solvent or medium. The high concentrations in FILs (around 1–2 M) lead to strong interactions between functional groups and ions; these interactions can play dominant roles in new IL-based functional materials. For example, magnetic ILs consisting of metal complexes have been reported.^16–18^ Hisamitsu *et al.* reported photo-functionalized ionic liquids with photon-up-conversion characteristics,^19,20^ with concentrated chromophores being a critical design in their FILs. Murray *et al.* reported conductive redox-active ILs (RAILs) that comprise metal complexes and viologens.^21–23^ In addition, Saielli *et al.* reported the thermal properties of various viologen-based ILs and ionic liquid crystals, which are correlated with their structure.^24–26^ These findings demonstrate the potential of viologens to be a potent basic component for developing and creating FILs.

Several applications of RAILs in electrochemical reactions and devices have also been reported; they are used as components in supercapacitors,^27,28^ redox flow batteries,^29^ and electrochromic devices,^30–32^ and as electron mediators for photochemical catalysts.^33^ Additional examples have been given in recent reviews.^34,35^ They highlight the extended utility of RAILs as a class of FILs. We note that, in most of the cases, functionalities of FILs originate from individual functional groups and ions.

Let us now consider the use of CT interaction in ILs to create a new class of FILs. Kato *et al.* first reported an ion pair formation between an imidazolium and iodide in a neat imidazolium-based IL ([BMIM][I]), resulting from CT interaction between them.^36^ They indicate that the Madelung potential of [BMIM][I] determines the stabilization of the CT complex. Ogura *et al.* reported CT interaction between pyridinium as an acceptor and iodide as a donor in neat pyridinium-based IL ([Epy][I]) at 100 °C.^37^ They found that the CT complex in the neat [Epy][I] shows a different photo-absorption band from the diluted solution in dichloromethane, and they pointed out the importance of CT interaction between cation and anion in the neat [Epy][I]. Aster and Vauthey studied the CT interaction between anions of ILs and electron accepting additives in a photo-induced electron transfer reaction between the species.^38^ They concluded that the ILs play an active role in such photochemical systems rather than being an inert medium. With the incorporation of CT interactions into ILs, we may create a new functionality that is not possessed by individual donors and acceptors, and thereby modulate the physicochemical properties of the materials. In this study, we aim to exploit the functionality of viologens to design FILs based on CT interaction. In this regard, CT interactions between donors and viologens have been applied for assembling molecules,^2,39^ photo-induced electron transfer reactions,^5–8,40,41^ alkali metal sensing,^42^ and thermochromism.^43^ These studies demonstrated the potential of incorporating CT interactions into viologen-based ILs to pave a new path in IL research. To the best of our knowledge, research into RAILs with externally introduced CT interaction has not been attempted.

Intermolecular and interionic interactions in ILs have been investigated to understand the relationship between the structures and dynamic transport properties such as viscosity and ionic conductivity. In these species, electrostatic, van der Waals, hydrogen bonding, dipole–dipole, and π–π interactions all play important roles in the formation of microstructures, leading to unanticipated transport features and functionalities.^44,45^ Understanding these strong and weak interactions will provide new insights into the transport properties and functionalities in ILs. In this regard, it is noteworthy that the effect of CT interaction in ILs on the physicochemical and electrochemical properties is presently unclear.

In this study, we present a “RAIL with CT interaction” (CT-IL) originating from two redox centers, a carbazole and viologen; it is based on an equimolar mixture of a viologen-based IL and a carbazole-based IL. As we shall see, in the CT-IL, the CT interaction between the carbazole and viologen modulates the physicochemical properties such as viscosity and ionic conductivity, as well as redox properties including redox reversibility of viologens. Our approach provides a convenient means for creating and tuning of CT-IL, with properties that are distinct from those of the components.

## 2. Experimental

**Scheme 1:**
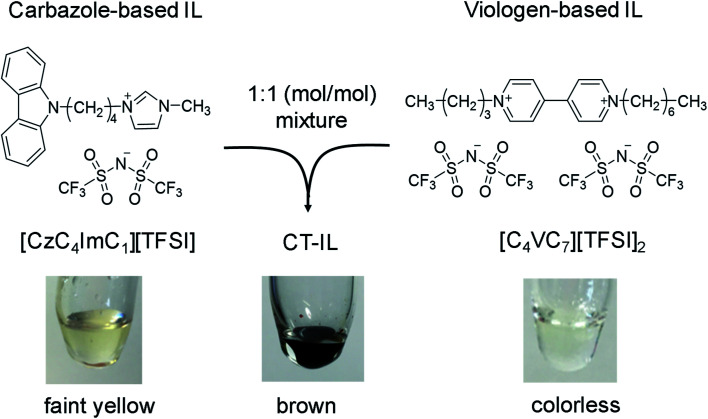
Preparation of a redox-active ionic liquid (RAIL) with charge transfer (CT) interaction from carbazole-based and viologen-based RAILs.

Chemical structures of the carbazole-based RAIL ([CzC_4_ImC_1_][TFSI]) and the viologen-based RAIL ([C_4_VC_7_][TFSI]_2_) in this study are shown in Scheme 1, with a photo of the combined CT-IL straightforwardly prepared from an equimolar mixture between them. Synthetic procedures of the RAILs, water content in the RAILs, and experimental setups are described in the ESI.† All measurements in this study were conducted in neat RAILs, namely without any solvent, unless otherwise mentioned. [C_4_VC_7_][TFSI]_2_ remains in a supercooled liquid state for a few hours at room temperature after it was heated above the melting point (52 °C).^46^ Although the melting points of [CzC_4_ImC_1_][TFSI] and CT-IL could not be determined by differential scanning calorimetry (DSC) because of the absence of any peak assignable to the melting point, their solidification was not observed at room temperature (see the DSC thermograms in Fig. S1†). Therefore, [C_4_VC_7_][TFSI]_2_, [CzC_4_ImC_1_][TFSI], and CT-IL can be handled as room temperature ILs.^32^

## 3. Results and discussion

### 3.1 Optical properties

#### 3.1.1 Absorption spectra

**Fig. 1:**
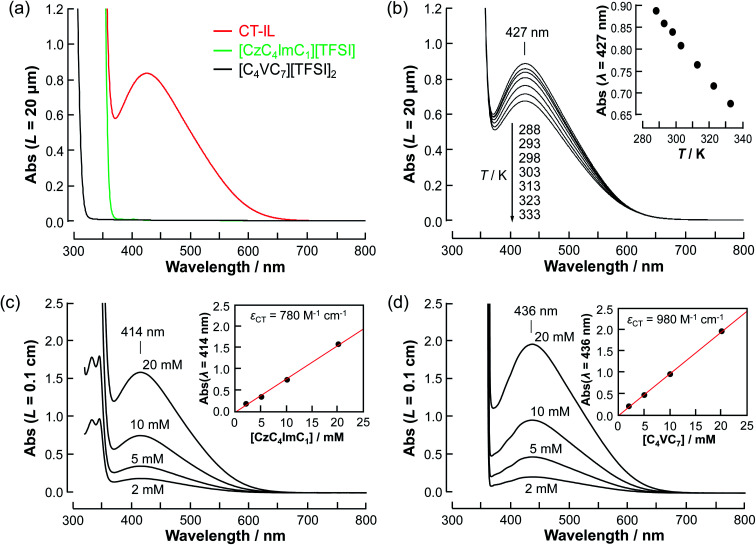
Absorption spectra of [CzC_4_ImC_1_][TFSI], [C_4_VC_7_][TFSI]_2_, and CT-IL. (a) The neat RAILs (20 µm optical path length) at 298 K. (b) The temperature-dependence of the neat CT-IL. The inset plot is the absorbance of the CT absorption at 427 nm wavelength as a function of the temperature. (c) *x* mM [CzC_4_ImC_1_][TFSI] (*x* = 2, 5, 10, 20) in [C_4_VC_7_][TFSI]_2_ at 297 K and (d) *y* mM [C_4_VC_7_][TFSI]_2_ in [CzC_4_ImC_1_][TFSI] (*y* = 2, 5, 10, 20) at 297 K (0.1 cm optical path length).

To investigate the CT characteristics of the RAILs, we recorded the absorption spectra of [CzC_4_ImC_1_][TFSI], [C_4_VC_7_][TFSI]_2_, and the CT-IL in the neat system (Fig. 1(a)). The neat [CzC_4_ImC_1_][TFSI] and [C_4_VC_7_][TFSI]_2_ have no absorption band over the visible region as well as their diluted acetonitrile solutions (Fig. S2†). For the neat CT-IL, a new broad absorption band at 427 nm was observed. The absorption band is attributable to the absorption by the CT complex between the carbazole and viologen.^7^ Specifically, our absorption spectrum is in good agreement with the CT absorption spectrum reported by Yonemura *et al.*^5^ for a diluted carbazole-tethered viologen, in terms of not only peak wavelength but also line width. Thus, the formation of additional species such as higher aggregates of the CT complex in CT-IL is unlikely. The CT absorption band of CT-IL at 427 nm monotonically decreased with increasing the temperature, holding the peak wavelength invariant. This further supports a simple equilibrium of 1 : 1 complex of carbazole and viologen, the same as in the diluted system. Taking these considerations into account, the temperature dependence can be explained by the association/dissociation equilibrium of the carbazole and viologen (Cz + V^++^ ⇌ CzV^++^).

As a first approximation, we do not consider carbazole–carbazole interaction and viologen–viologen interaction. The total concentration (*c*_0_) of the carbazoles and viologens of CT-IL here was 0.92 M in the 1 : 1 (molar ratio) mixture (*c*_0_ = [Cz]_0_ = [V^++^]_0_) (see the ESI†). The concentration of the CT complex (*c*_CT_) can be described by the thermodynamic equilibrium constant.1
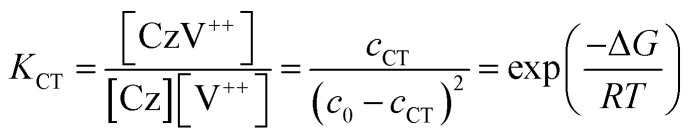


The molar absorption coefficient (*ε*_CT_) of the CT complex between carbazoles and viologens was reported to be around 400 ± 70 M^−1^ cm^−1^ at 420 nm.^7^ However, the absorption coefficient is invalid in our CT-IL because if we use it as an approximated value, the concentration of the CT complex is estimated to be over 1.0 M at 298 K with the Lambert–Beer relation (Abs_CT_ = *ε*_CT_*Lc*_CT_) despite *c*_0_ = 0.92 M. Usually, those parameters can be determined from the concentration dependence of the donor and acceptor in the same solvent. However, CT-IL here consists of only the donor ([CzC_4_ImC_1_][TFSI]) and acceptor ([C_4_VC_7_][TFSI]_2_) without solvent. That is, the donor and the acceptor themselves play the role of a medium as well. Thus, the concentration and the fraction changes give rise to the polarity changes of the solvent (medium). To approximate *ε*_CT_ and *K*_CT_ of the CT-IL, we prepared two sets of diluted solutions, (1) *x* mM [CzC_4_ImC_1_][TFSI] diluted by [C_4_VC_7_][TFSI]_2_ (*x* = 2, 5, 10, 20) and (2) *y* mM [C_4_VC_7_][TFSI]_2_ diluted by [CzC_4_ImC_1_][TFSI] (*y* = 2, 5, 10, 20), and measured the absorption spectra. Note that two sets of solutions satisfy the following concentration relationship: [CzC_4_ImC_1_] (*x* mM, solute) ≪ [C_4_VC_7_] (1.8 M, solvent) in system (1) and [C_4_VC_7_] (*y* mM, solute) ≪ [CzC_4_ImC_1_] (2.7 M, solvent) in system (2). Besides, the polarity of [C_4_VC_7_][TFSI]_2_ and [CzC_4_ImC_1_][TFSI] could be close to that for the CT-IL. Fig. 1(c) and (d) show absorption spectra of the CT complex in systems (1) and (2). The absorbances were proportional to the concentration of the diluted species. In these cases, *i.e.* with a large excess of C_4_VC_7_ or CzC_4_ImC_1_, the absorbance of the CT complex can be described as follows.2
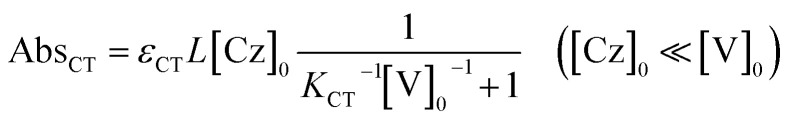
3
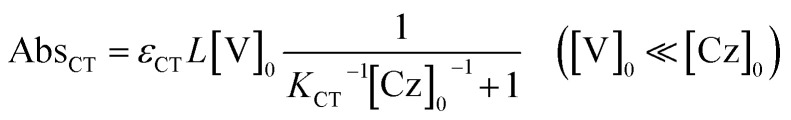
where [Cz]_0_ and [V]_0_ are the starting concentration of the carbazole and viologen. The concentration of the CT complex can be approximated to be that for the diluted [Cz]_0_ or [V]_0_ when *K*_CT_^−1^[V]_0_^−1^ ≪ 1 namely *K*_CT_ ≫ 0.56 M^−1^ for eqn (2) (system (1)) and *K*_CT_^−1^[Cz]_0_^−1^ ≪ 1 namely *K*_CT_ ≫ 0.37 M^−1^ for eqn (3) (system (2)). Under the assumptions of *K*_CT_^−1^[V]_0_^−1^ ≪ 1 and *K*_CT_^−1^[Cz]_0_^−1^ ≪ 1, the molar absorption coefficients of the CT complex were determined from the slopes of the obtained straight lines in Fig. 1(c) and (d) to be 780 and 980 M^−1^ cm^−1^. Those values greater than the previously reported value^7^ are possibly due to the polarity difference of the medium. Furthermore, the peak wavelength of the CT absorption in CT-IL (427 nm) locates between the wavelengths in [C_4_VC_7_][TFSI]_2_ (414 nm) and [CzC_4_ImC_1_][TFSI] (436 nm). The polarity of the CT-IL, *i.e.*, with an equimolar mixture of [CzC_4_ImC_1_][TFSI] and [C_4_VC_7_][TFSI]_2_, can be in between the polarities of [CzC_4_ImC_1_][TFSI] and [C_4_VC_7_][TFSI]_2_. The concentration of the CT complex (*c*_CT_) in CT-IL is 0.48 M at 298 K with an assumption of *ε*_CT_ to be 880 M^−1^ cm^−1^ in the CT-IL, which is taken as an average of the absorption coefficients in Fig. 1(c) and (d). Around 50 mol% carbazole and viologen in the CT-IL achieve the CT complexation. Its thermodynamic parameters are Δ*G* = −2.2 kJ mol^−1^, Δ*H* = −13.9 kJ mol^−1^, and Δ*S* = +39.4 J mol^−1^ K^−1^ (see Fig. S3 and Table S1†). The theoretical evaluation using density functional theory (DFT) showed Δ*G* = −3.6 kJ mol^−1^, Δ*H* = −49.8 kJ mol^−1^ and Δ*S* = +155 J mol^−1^ K^−1^ at 298 K with a model compound of CzV^++^ in an IL medium (see the ESI†). The theoretical evaluation provided reasonable Δ*G* for CT-IL, although the assumption of the isolated CT complex surrounded by the continuous IL dielectric medium was far different from the real system.

#### 3.1.2 Fluorescence quenching

Emissions of 10 µM [CzC_4_ImC_1_][TFSI] in acetonitrile and neat [CzC_4_ImC_1_][TFSI] were observed at around 360 nm when excited at 293 nm and 430 nm when excited at 328 nm (Fig. S4†). The emission of 10 µM [CzC_4_ImC_1_][TFSI] in acetonitrile is attributed to the monomolecular fluorescence of carbazole. The emission of neat [CzC_4_ImC_1_][TFSI] was located at longer wavelengths with a vibration structure. The origin of the emission in neat [CzC_4_ImC_1_][TFSI] is not clear but it is beyond our focus in this study. On the other hand, we observed no emission in CT-IL system. This can be attributed primarily to the fast fluorescence quenching by photo-induced electron transfer from the excited carbazole to the viologen.^5,47^ As can be seen above, neat CT-IL shows rather different optical properties from individual RAILs and the diluted CT-IL in acetonitrile.

### 3.2 Viscosity and ionic conductivity

#### 3.2.1 Arrhenius and Vogel–Fulcher–Tammann analysis

**Fig. 2:**
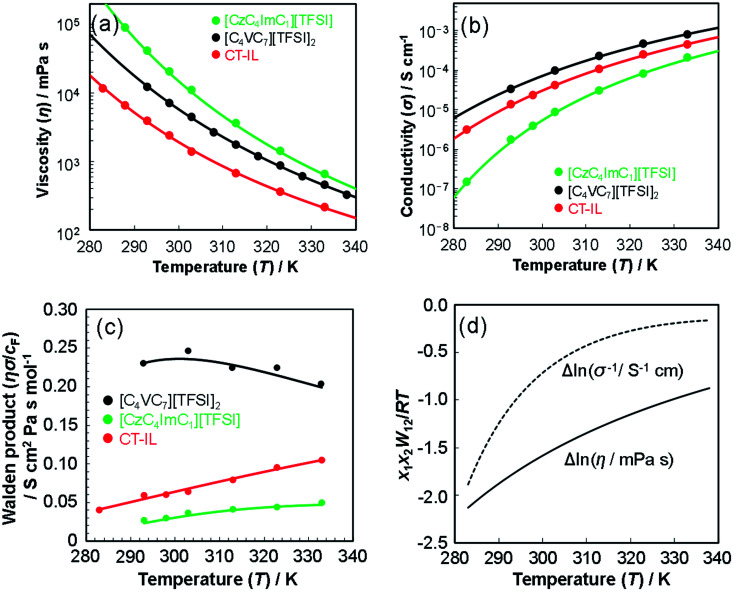
Temperature-dependent transport properties of [CzC_4_ImC_1_][TFSI], [C_4_VC_7_][TFSI]_2_, and CT-IL. (a) Viscosity, (b) conductivity, (c) Walden product of RAILs, and (d) interaction parameters *W*_12_ of CT-IL evaluated using the Grunberg–Nissan model with neat [CzC_4_ImC_1_][TFSI] and [C_4_VC_7_][TFSI]_2_. Solid lines in (a)–(c) are represented by fitted VTF lines. At the Walden product (c), the conductivities were divided by the formal concentrations (*c*_F_) as constant values in the whole temperature range, 2.7 M for [CzC_4_ImC_1_][TFSI] (2.7 M CzC_4_Im^+^C_1_ and 2.7 M TFSI^−^), 1.8 M for [C_4_VC_7_][TFSI]_2_ (1.8 M C_4_V^++^C_7_ and 3.6 M TFSI^−^) and 0.92 M for CT-IL (0.92 M CzC_4_Im^+^C_1_, 0.92 M C_4_V^++^C_7_, and 2.7 M TFSI^−^).

Temperature dependences of the viscosity and the ionic conductivity of [CzC_4_ImC_1_][TFSI], [C_4_VC_7_][TFSI]_2_, and CT-IL are shown in Fig. 2(a) and (b). The viscosity increases in the order of CT-IL < [C_4_VC_7_][TFSI]_2_ < [CzC_4_ImC_1_][TFSI] at temperatures between 293 K and 333 K, while the ionic conductivity shows a different order of [CzC_4_ImC_1_][TFSI] < CT-IL < [C_4_VC_7_][TFSI]_2_ from the viscosity. The viscosity of CT-IL is the lowest among the three RAILs, while the conductivity of CT-IL is not the highest among them. To rationalize the trends, the activation energy was evaluated using the Arrhenius equations (eqn (4)), and the approximated interpolation equations (eqn (5)) were expressed by the Vogel–Fulcher–Tammann (VFT) formalism:4
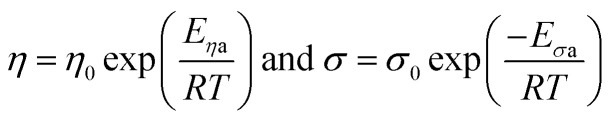
5
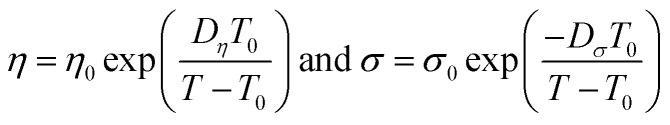


The best fit parameter-sets are listed in Table 1. The Arrhenius plot of the viscosities and ionic conductivities (Fig. S6†) shows a little deviation from straight lines. The coefficient of determination (*R*^2^) for the viscosity of CT-IL is 0.9914, and that for the ionic conductivity of [CzC_4_ImC_1_][TFSI] is 0.9761 on the basis of the Arrhenius equation. Taking these into consideration, we can see that these RAILs obey VFT formalism resulting in better *R*^2^ values over 0.999.

**Table 1:** Fitting results of the viscosity and ionic conductivity data

RAILs	Arrhenius parameter set			Vogel–Fulcher–Tammann parameter set			
	*η* = *η*_0_ exp(*E*_*η*a_/*RT*)			*η* = *η*_0_ exp(*D*_*η*_*T*_0_/(*T* − *T*_0_))			
	*σ* = *σ*_0_ exp(−*E*_*σ*a_/*RT*)			*σ* = *σ*_0_ exp(−*D*_*σ*_*T*_0_/(*T* − *T*_0_))			
	*E*_*η*a_/kJ mol^−1^	ln(*η*_0_/Pa s)	*R* ^2^	*D*_*η*_ *T*_0_/K	*T*_0_/K	ln(*η*_0_/Pa s)	*R* ^2^
	*E*_*σ*a_/kJ mol^−1^	ln(*σ*_0_/S cm^−1^)		*D*_*σ*_ *T*_0_/K		ln(*σ*_0_/S cm^−1^)	
[C_4_VC_7_][TFSI]_2_^a^	63.1	−23.58	0.9941	1147	193.9	−9.053	0.9997
	64.2	16.21	0.9917	659.0	219.8	−1.303	0.9999
[CzC_4_ImC_1_][TFSI]	87.0	−32.03	0.9952	1413	194.7	−10.66	0.9999
	109	31.42	0.9761	774.5	230.3	−1.005	0.9996
CT-IL	62.8	−24.44	0.9914	880.8	200.6	−8.217	0.9995
	77.0	20.36	0.9896	1019	203.8	0.1992	0.9997

a Viscosity data and the VTF parameters were taken from ref. 46.

The activation energy (*E*_*η*a_) for the viscosity follows the order CT-IL ≈ [C_4_VC_7_][TFSI]_2_ < [CzC_4_ImC_1_][TFSI]. In comparison, activation energy (*E*_*σ*a_) for the conductivity shows a different order [C_4_VC_7_][TFSI]_2_ < CT-IL < [CzC_4_ImC_1_][TFSI]. This suggests that the activation processes of viscosity and conductivity are different. This tentative conclusion is also supported by the difference in the trends for *D*_*η*_*T*_0_ and *D*_*σ*_*T*_0_ in VTF formalism, as interpreted from the activation energy, *i.e.*, CT-IL < [C_4_VC_7_][TFSI]_2_ < [CzC_4_ImC_1_][TFSI] for the viscosity and [C_4_VC_7_][TFSI]_2_ < [CzC_4_ImC_1_][TFSI] < CT-IL for the conductivity. The activation energy (*E*_*η*a_) of the viscosity of CT-IL is reduced by mixing the two RAILs. To examine the characteristics of the ionic transportation, we use the Walden analysis and the Grunberg–Nissan^48–51^ analysis below.

#### 3.2.2 Walden analysis

For the investigation of transportation parameters of ILs, the Walden relation (*ησ* = constant) has often been used, even though it originally holds in a diluted ionic solution. The Walden product, *ησ* or *ησ*/*c*, remains constant, independent of the temperature in the absence of inter-ionic interactions. Fig. 2(c) shows the temperature dependence of the Walden product of the RAILs obtained by using the formal concentration (*c*_F_), while the full Walden plot is shown in Fig. S7.† The density of ILs and thus *c*_F_ of the ions slightly decreased with increasing temperature because of volumetric expansion. However, the changes are negligibly small.^5,31^ Therefore, we assumed the formal concentrations are temperature-independent constants for the Walden analysis.

In comparison, the Walden product of CT-IL increased with increasing temperature, while that of [CzC_4_ImC_1_][TFSI] remains nearly constant with a slight increase, and that of [C_4_VC_7_][TFSI]_2_ slightly decreases. To put these results into perspective, the Walden product of a conventional IL [BMIM][BF_4_] remains almost constant.^52^ In CT-IL, the ionic association and the CT complex formation can be deeply involved in the transport properties. Therefore, the temperature dependent Walden product of CT-IL is attributable to both equilibria: association and dissociation of (1) ionic components (between the viologen, imidazolium, and TFSI), and (2) CT complex (between the carbazole and viologen).

#### 3.2.3 Grunberg–Nissan analysis

As an alternative method of analysis, we evaluated the viscosity of binary mixtures using the Grunberg–Nissan model.^48–51^6
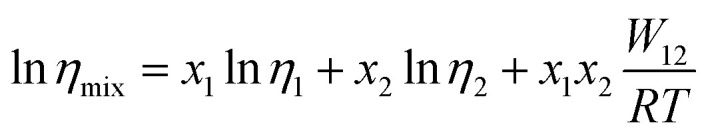
where *η*_1_, *η*_2_, and *η*_mix_ are the viscosities of compounds 1, 2, and their mixture, respectively; *x*_1_ and *x*_2_ are the molar fractions, and *W*_12_ is the interaction parameter between compounds 1 and 2. If the interaction parameter *W*_12_ is negligibly small, the viscosity of the mixture is a fraction-weighted logarithmic average, that is, it signifies a non-associated liquid. When *W*_12_ is non-zero positive/negative, the viscosity of the mixture should be greater/smaller than those of the individual compounds. The experimentally obtainable interaction parameters for viscosity, and those for ionic conductivity, in the analogy to the viscosity, can be evaluated using the following expression.7
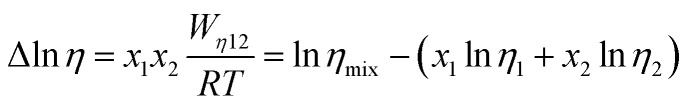
8
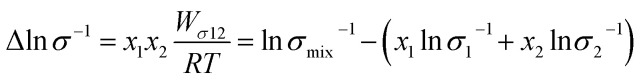


Fig. 2(d) shows the temperature dependence of Δln *η* and Δln *σ*^−1^ for CT-IL, obtained using the continuous data interpolated with the VTF formula of the viscosity and conductivity as well as the fitted parameters in Table 1. The interaction parameters were negative, and the magnitudes of both viscosity and conductivity decreased monotonically with increasing the temperature. The curvatures of Δln *η* and Δln *σ*^−1^ are different, where Δln *σ*^−1^ shows more convex behavior.

**Fig. 3:**
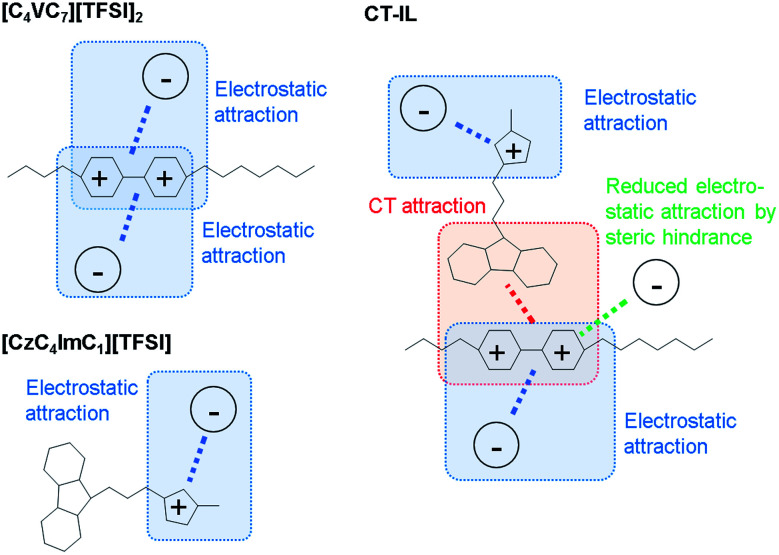
Illustration of attractions between the cations (imidazolium and viologen) and the anion (TFSI).

In the CT-IL system, two kinds of equilibria should be considered: (1) electrostatic cation–anion pair association and dissociation and (2) formation of the CT complex. In ionic liquids, electrostatic attraction between anions and cations dominates the attractive interaction that leads to the association of cations and anions (Fig. 3(a) and (b)). The associations can be examined in terms of ionicity by the impedance technique and nuclear magnetic resonance.^53^ The decreased viscosity in CT-IL upon mixing [CzC_4_ImC_1_][TFSI] and [C_4_VC_7_][TFSI]_2_ can be attributed to decreasing electrostatic attraction because of the formation of the CT complex between the carbazole and the viologen (Fig. 3(c)). Our DFT calculation supports the decrease of the interionic attraction. We employed a fragment molecular orbital (FMO) calculation to evaluate the CT interaction which contributes to the charge donation from the carbazole to the viologen. We found that the carbazole donates 0.163 electrons to the viologen resulting in decreasing its cationic charge, *i.e.* the formal charge can be depicted as Cz^+0.163^V^+1.837^. Besides, the attraction enthalpy difference between the viologen and TFSI is 63.4 kJ mol^−1^ with carbazole and 65.9 kJ mol^−1^ without carbazole (geometries of the contact ionic pairs are shown in Fig. S17†). Thus, the CT attraction can weaken the electrostatic attraction between the viologen and TFSI. Therefore, we may expect that decreased viscosity would lead to increasing ionic conductivity. However, the ionic conductivity of CT-IL was in fact smaller than that of [C_4_VC_7_][TFSI]_2_.

To explain the smaller ionic conductivity of CT-IL than [C_4_VC_7_][TFSI]_2_, we note that the actual concentration of free ionic species is important for ionic conductivity. Formation of the CT complex would lead to decreasing concentration of the free dicationic viologens and suppressing the movement and migration of the viologens and imidazoliums linked with carbazole. Because increasing temperature leads to dissociation of both the ions and the CT complex, this results in a steeper temperature dependence on Δln *σ*^−1^ than on Δln *η*.

The viscosity and ionic conductivity of those RAILs can be affected by the temperature-dependent microstructural and dynamic heterogeneities of the ILs.^44,45^ For our [CzC_4_ImC_1_][TFSI] and [C_4_VC_7_][TFSI]_2_ systems, however, no data suggest the presence of the such phase-separated structures in these RAILs.

### 3.3 Redox properties

#### 3.3.1 Brief overview of the redox of the carbazole and viologen in CT-IL

**Fig. 4:**
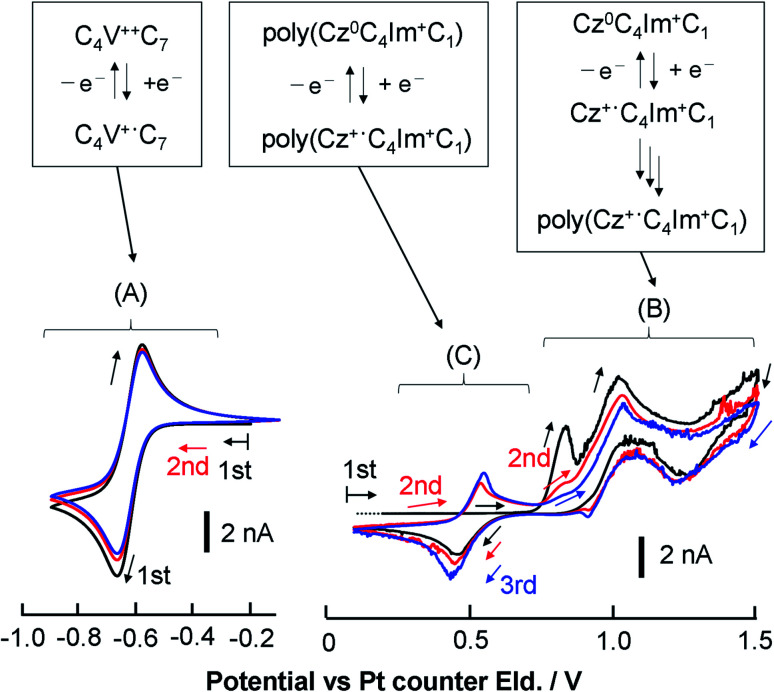
Overview of the electrochemical reaction of neat CT-IL. Multiple CV scans of neat CT-IL with a Au microelectrode at 333 K and 50 mV s^−1^ in the viologen region and the carbazole region (black for the 1st, red for the 2nd, and blue for the 3rd scan). The capital alphabets denote the potential regions: (A) redox of the viologen; (B) redox of carbazole and the electro-polymerization; (C) redox of the electro-polymerized carbazole (poly(CzC_4_ImC_1_)).

CVs of the neat CT-IL at 333 K and 50 mV s^−1^ are shown in Fig. 4. A reversible redox wave of the viologen was observed around −0.6 V (region A). Anodic currents originating from the oxidation of carbazoles were found around +0.7 to +1.5 V (region B). Although no anodic current was found around +0.5 V during the first positive scan, a redox pair in that region appeared in the second and subsequent scans resulting from the redox reaction of electro-polymerized carbazole produced by the oxidation of monomeric carbazole in potential region B.^54,55^ The similar responses of carbazole and poly-carbazole were likewise seen in neat [CzC_4_ImC_1_][TFSI] (Fig. S8†). However, the redox responses of the carbazole and the electropolymerized carbazole were complicated and the analysis was difficult. Therefore, we focused our attention on the redox response of the viologen.

#### 3.3.2 Redox reversibility of the viologen: the Nernstian and the interacting two-redox site models

**Fig. 5:**
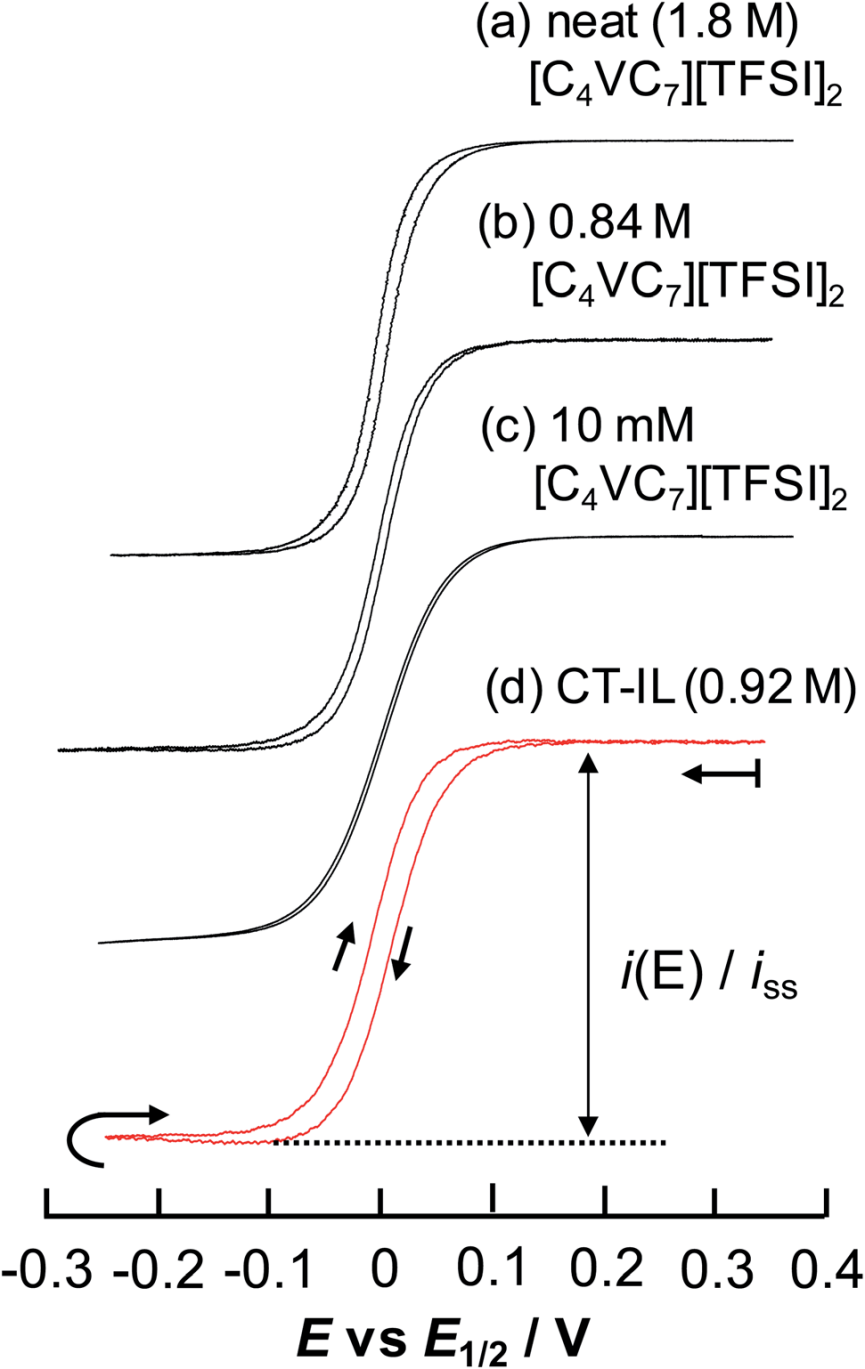
Steady-state CVs of [C_4_VC_7_][TFSI]_2_ at various concentrations at 333 K with a Au-UME using a slow scan rate within the reduction region of viologens. (a) Neat [C_4_VC_7_][TFSI]_2_ at 0.1 mV s^−1^, (b) 0.84 M [C_4_VC_7_][TFSI]_2_ in [BMIM][TFSI] (1 : 2 molar ratio mixture of [C_4_VC_7_][TFSI]_2_ and [BMIM][TFSI]) at 0.5 mV s^−1^, (c) 10 mM [C_4_VC_7_][TFSI]_2_ in [BMIM][TFSI] at 1 mV s^−1^, and (d) CT-IL at 0.5 mV s^−1^. The currents were normalized by the steady-state limiting current. Nernstian parameters related to the CVs are listed in Table 2.

CVs of the viologen in region A in Fig. 4 produced peaked diffusion-limited waveforms due to semi-infinite planar diffusion to the electrode surface. For analytical evaluation of electrochemical reversibility of the viologen, we investigated CVs of the viologens at some concentrations with slow scan rates at which the CVs show a sigmoidal waveform. Reducing the scan rate brought the CVs to a steady state without current and shape changes. Thus, the CVs can be regarded as electrochemically reversible responses. Fig. 5 shows steady-state CVs showing sigmoidal waveforms at 333 K for four systems: neat [C_4_VC_7_][TFSI]_2_, 0.84 M [C_4_VC_7_][TFSI]_2_ in [BMIM][TFSI], 10 mM [C_4_VC_7_][TFSI]_2_ in [BMIM][TFSI], and CT-IL. Although hysteresis was found due to a contribution of the planar diffusion (see the ESI†), all samples showed sigmoidal waveforms, whose forward and reverse scan curves were superimposable by shifting the potential axis less than 19 mV. That is, the systems again showed electrochemically reversible CVs whose line shapes did not vary at lower scan rates. However, the slope of the CVs at *E* = *E*_1/2_ was dependent on the system. The three systems, neat [C_4_VC_7_][TFSI]_2_, 0.84 M [C_4_VC_7_][TFSI]_2_ in [BMIM][TFSI] and CT-IL showed a steeper slope than 10 mM [C_4_VC_7_][TFSI]_2_ diluted in [BMIM][TFSI].

In principle, when the electrochemical reaction is described by a simple electrochemical equilibrium (Ox + e^−^ ⇄ Red) of a redox couple and the redox species are not interacting with each other, the Nernst equation is established and can provide the concentration ratio of the redox species on the electrode surface [Ox]_surf_/[Red]_surf_. In that situation, a steady-state voltammogram can be described by the ideal Nernst response eqn (9) under the assumption of equal diffusion coefficients *D*_Ox_ = *D*_Red_ with *n*_app_ = 1.9
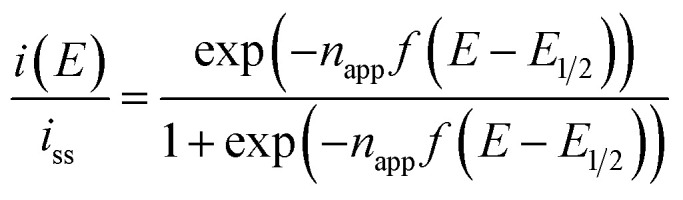
where *f* = *F*/*RT*, *i*_ss_ is the steady-state limiting current whose sign is defined so that the cathodic current goes negative, *E*_1/2_ is the half-wave potential and *n*_app_ is an apparent number of electrons representing the redox equilibrium. Here, *n*_app_ can take a non-integer value to appropriately describe the redox characteristics of CVs. In fact, the diluted system of 10 mM [C_4_VC_7_][TFSI]_2_ in [BMIM][TFSI] produced a Nernstian CV with *n*_app_ = 1.00. However, the other systems with higher concentrations of the viologens indeed produced Nernstian CVs with greater *n*_app_ than unity, *i.e.* the slope of the CV at *E* = *E*_1/2_ is steeper than that with *n*_app_ = 1 (see Table 2). It implies that the reduced viologens are interacting attractively. The attractive interaction brings about a situation where the reduction potential of an Ox species close to a Red species shifts positively when the Ox species accepts an electron from the electrode to be the Red species. The schematic representation of the attractive reaction can be proposed as follows.V^2+^ + e^−^(eld) ⇄ V^+^˙ (step 1)V^+^˙ + V^2+^(neighbor) ⇄ V^+^˙⋯V^2+^ (step 2)V^+^˙⋯V^2+^ + e^−^(eld) ⇄ V^+^˙⋯V^+^˙ (step 3)

**Table 2:** Analyzed parameters of steady state current in the sigmoidal CVs of RAILs consisting of [C_4_VC_7_][TFSI]_2_ at 333 K (Fig. 5)

RAILs	Concentration of C_4_VC_7_/M	*i*_ss_/nA	*D*_app_/cm^2^ s^−1^	*n*_app_	Δ*E* = *E*_1_ − *E*_2_/mV	Viscosity (*η*)/mPa s	Conductivity (*σ*)/S cm^−1^
(a) Neat [C_4_VC_7_][TFSI]_2_	1.8	−8.38	2.4 × 10^−8^	1.60^a^	−55.6^a^	456	8.0 × 10^−4^
				1.66^b^	−64.6^b^		
(b) Mixture of [C_4_VC_7_][TFSI]_2_ and [BMIM][TFSI] (1 : 2 molar ratio)	0.84	−13.0	8.0 × 10^−8^	1.43^a^	−20.0^a^	N/A	N/A
				1.39^b^	−15.6^b^		
(c) Diluted [C_4_VC_7_][TFSI]_2_ in [BMIM][TFSI]	0.010	−0.466	2.4 × 10^−7^	1.00	+39.9	15.4^c^	1.1 × 10^−2^^d^
(d) CT-IL	0.92	−1.85	1.0 × 10^−8^	1.22^a^	+13.2^a^	214	4.5 × 10^−4^
				1.23^b^	+7.6^b^		

a Forward scan.

b Reverse scan.

c Pure [BMIM][TFSI] from ref. 58 using the VFT equation.

d Pure [BMIM][TFSI] from ref. 59 using the VFT equation.

Step 3 is the dimerization reaction of the reduced viologen as discussed below. If Δ*G* in step 3 is negatively large and the reactions from step 1 to step 3 are regarded as a sequential redox process with two redox sites, we can approximately describe the reaction as a two-consecutive one-electron transfer process. In that case, the reduction potential difference Δ*E* = *E*_1_ − *E*_2_, the first and second reduction potentials *E*_1_ and *E*_2_, can negatively increase with increasing the attractive interaction which means the system prefers the dimerized viologens (V^+^˙⋯V^+^˙). The two steps of the reduction reaction eventually cannot be distinguished from each other but seen to be coalescent apparently resulting in an indistinguishable two-electron reduction reaction. Under the assumption that diffusion coefficients of all species involved in the reaction are the same, the steady-state voltammogram can be described as follows.^56,57^10
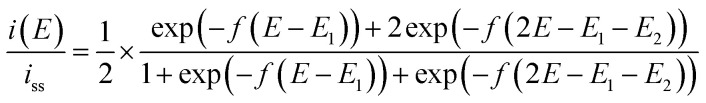
where *E*_1_ and *E*_2_ are the macroscopic redox potentials for the two redox steps. Here we defined an interaction parameter, Δ*E* = *E*_1_ − *E*_2_, which reflects an inter-site interaction in the redox reaction as described above. If there is no inter-site interaction in the system, *i.e.* redox potentials of all steps are equal (*ε*_1_ = *ε*_2_ = *ε*_3_ = *ε*_4_ in ref. 56), it turns out that Δ*E* = (2 ln 2)*RT*/*F* = +39.8 mV at 333 K and *n*_app_ becomes unity. If the inter-site interaction is repulsive/attractive, *i.e.* one reduced site discourages/encourages the reduction reaction on another site, then Δ*E* > +39.8 mV for repulsive and Δ*E* < +39.8 mV for attractive interaction.

The results from analyzing the CVs in Fig. 5 are listed in Table 2. The diluted solution of 10 mM [C_4_VC_7_][TFSI]_2_ exhibited a typical Nernstian CV with *n*_app_ = 1 and Δ*E* = +39.8 mV, which indicates the absence of interaction between viologens. Other viologen systems exhibit Δ*E* < +39.8 mV and *n*_app_ > 1, which suggest attractive interaction between viologens. A possible attraction of viologens was the dimerization between reduced species (V^+^˙ + V^+^˙ ⇌ V^+^˙⋯V^+^˙). In relation to such dimerization, the dimerization constants of viologens (*K*_D_ = [V^+^˙⋯V^+^˙]/[V^+^˙]^2^) in water and organic solvent systems are typically 10^3^ to 10^5^ M^−1^ (ref. 60–62) with the corresponding Gibbs energy changes being −18.8 to −29.1 kJ mol^−1^.^61^ In our previous electroreflectance spectroscopy experiments, we have also observed, in the steady-state CV, intramolecular dimerization of a diluted bis-viologen in water as a result of attractive interaction.^57^ In addition, we have also reported electrochromic devices based on viologen-based RAILs, with dimerizations being observed as a result of highly concentrated viologens.^31,33^

The concentrated viologens in this study reached around 1–2 M, and we therefore deem dimerization as induced by reduction possible. The order of −Δ*E* and *n*_app_ values is (c) 10 mM [C_4_VC_7_][TFSI]_2_ (no attraction) < (d) CT-IL < (b) 0.84 M [C_4_VC_7_][TFSI]_2_ < (a) neat [C_4_VC_7_][TFSI]_2_. Neat [C_4_VC_7_][TFSI]_2_ (a) exhibited the greatest *n*_app_ and smallest Δ*E* because of its highest viologen concentration. Interestingly, the concentration of C_4_VC_7_ in (d) CT-IL (0.92 M) is similar to that for (b) 0.84 M [C_4_VC_7_][TFSI]_2_, while the *n*_app_ and Δ*E* values were quite different. This can be explained by the CT complexation between the viologen and carbazole, which weakened the inter-viologen dimerization. Three such mechanisms were plausible. One mechanism is that CT complexation weakens the attraction of free V^++^ species to V^+^˙; the other mechanism is that the reduction potential of the CT complex (CzV^++^) might be more negative than that for free V^++^, which then results in the increase of Δ*E* greater than the value in 0.84 M [C_4_VC_7_][TFSI]_2_.

The diffusion coefficients can be evaluated by the steady state limiting current (*i*_ss_) with the Saito equation (*i*_ss_ = 4*nFrcD*) for microelectrodes.^63^ The migration effect in the absence of the supporting electrolyte system should not be ignored but the contribution to the limiting current in our system could be small.^64^ Therefore, we directly used the observed limiting current to evaluate the diffusion coefficient without any migration correction. The diffusion coefficients of the viologen (*D*_V_) in [C_4_VC_7_][TFSI]_2_ and CT-IL at 60 °C are, respectively, 2.4 × 10^−8^ and 1.0 × 10^−8^ cm^2^ s^−1^ in the CVs of Fig. 5. The diffusion coefficients of the concentrated system can be explained by the Dahms–Ruff model (*D*_V_ = *D*_phys_ + *D*_ex_)^65,66^ as the sum of physical diffusion and electron hopping processes. Although we cannot clarify the details of the process in the redox reaction at this stage, we need to mention the relation between the diffusion coefficient in the CVs and the transport properties such as viscosity and ionic conductivity. Interpretation of these diffusion coefficients should be presently made for two limiting cases.

##### In the case of *D*_phys_ ≫ *D*_ex_

The above-mentioned magnitude relation, *D*_V_([C_4_VC_7_][TFSI]_2_) > *D*_V_(CT-IL), cannot be simply explained by the magnitude relation of the viscosities (456 and 214 mPa s) with the Stokes–Einstein equation (*D*_phys_ = *k*_B_*T*/6π*aη*, *a* is the molecular radius) for the physical diffusion process. To explain the diffusion coefficients within the Stokes–Einstein framework, we can write the following relation *a*_V,CT-IL_/*a*_V,C_4_VC_7__ = *D*_V,CT-IL_*η*_CT-IL_/*D*_V,C_4_VC_7__*η*_C_4_VC_7__ and we obtained *a*_V,CT-IL_/*a*_V,C_4_VC_7__ = 5.1. This can be translated by association of C_4_VC_7_ with not only CzC_4_ImC_1_ but also TFSI anions in the CT-IL.

##### In the case of *D*_ex_ ≫ *D*_phys_

The diffusion coefficient electron hopping can be described by the Ruff equation (*D*_ex_ = *δ*^2^*k*_ex_*c*/6).^66,67^ Center-to-center distances between viologens in [C_4_VC_7_][TFSI]_2_ and CT-IL were estimated using the equation *δ* = (*N*_A_*c*)^−1/3^ (where *N*_A_ is the Avogadro constant and *c* is the molar concentration), respectively, to be 0.97 and 1.2 nm. Thus, the electron hopping rate constants *k*_B_ were, respectively, 8.5 and 4.6 ×10^6^ M^−1^ s^−1^. The electron hopping rate constant *k*_ex_ in viscous solution can be described by an atmosphere relaxation model^68,69^ which is an ion-pairing process to hold the electroneutrality in the electron hopping process. The magnitude relation of the diffusion coefficients agrees with that of ionic conductivities not that of the viscosities.

The details of the relationship among the transportation properties require further experiments such as diffusion ordered spectroscopy (DOSY)-NMR. We will perform and report it elsewhere in the future. Although the detailed mechanism of the redox process is at present not clear, what is clear is that CT interaction can notably modulate the physicochemical properties of ILs.

## 4. Conclusions

We demonstrated CT interactions in an IL produced by equimolar mixing of a carbazole-based ([CzC_4_ImC_1_][TFSI]) and a viologen-based ([C_4_VC_7_][TFSI]_2_) RAIL. The existence of a CT complex between the carbazole and viologen was confirmed by UV-vis absorption spectra, which neither [CzC_4_ImC_1_][TFSI] nor [C_4_VC_7_][TFSI]_2_ exhibited. The decrease of absorption with increasing temperature suggested the CT complex dissociation with increasing temperature. Transport properties are modulated by the new interaction; we found the viscosity and ionic conductivity of CT-IL to be different from those of the individual RAILs. The viscosity of CT-IL was smaller than those of [CzC_4_ImC_1_][TFSI] and [C_4_VC_7_][TFSI]_2_. Likewise, the ionic conductivity of CT-IL was also smaller than those of [CzC_4_ImC_1_][TFSI] and [C_4_VC_7_][TFSI]_2_. CV with a microelectrode showed that the redox response of the viologen was also modulated by CT interaction with the carbazoles. The apparent number of electrons involved in the redox reaction was larger than unity in concentrated solutions of [C_4_VC_7_][TFSI]_2_, indicating an attractive interaction between the redox species in accordance with a two-site redox model. In turn, this implies that dimerization of reduced viologens occurred much faster than the CV timescale. In comparison, CT-IL showed less attractive behavior than neat [C_4_VC_7_][TFSI]_2_, because CT complexation between the viologens and carbazoles suppresses viologen dimerization. Overall, the introduction of CT interaction into RAILs changes a wide range of chemical and physical properties. Thus, it represents a useful design tool for expanding the scope of FILs; a deep understanding of the relationship between the structures and the functions would be beneficial in advancing such systems.

## Author contributions

H. Tahara conceived and designed this study. S. Yonemori and S. Yamamoto synthesized the compounds. H. Tahara performed viscosity and spectroscopic measurements. H. Tahara, Y. Tanaka, and S. Yamamoto performed electrochemical measurements. H. Tahara and C. Bun performed the quantum chemical calculations. H. Tahara and T. Sagara analyzed the data. H. Tahara produced the manuscript. H. Murakami, C. Bun and T. Sagara checked and modified the manuscript.

## Funding sources

This work was supported by a Japan Society for the Promotion of Science (JSPS) Grant-in-Aid for Young Scientists (B) no. 26810052 (H. T.), Grant-in-Aid for Scientific Research (C) no. 20K05649 (H. T.), and the Hattori Hokokai Foundation (H. T.).

## Conflicts of interest

There are no conflicts to declare.

## Supplementary Material

SC-012-D0SC06244H-s001
