# Supplementary material for: A redox-active ionic liquid manifesting charge-transfer interaction between a viologen and carbazole and its effect on the viscosity, ionic conductivity, and redox process of the viologen
Source: Chem Sci. 2021 Feb 18;12(13):4872–82. doi: 10.1039/d0sc06244h (PMC8179547; doi:10.1039/d0sc06244h)
Supplement: SC-012-D0SC06244H-s001 [file SC-012-D0SC06244H-s001.pdf]

Electronic Supplementary Material (ESI) for Chemical Science.
This journal is © The Royal Society of Chemistry 2021

Supplementary Information

A redox-active ionic liquid manifesting charge-transfer interaction between a viologen and carbazole and its effect on the viscosity, ionic conductivity, and redox process of the viologen

Hironobu Tahara,[a] Yudai Tanaka,[a] Shoko Yamamoto,[a] Shigeki Yonemori,[b] Bun Chan,[a] Hiroto Murakami,[a] and Takamasa Sagara[a*]*

a Graduate School of Engineering, Nagasaki University 1-14 Bunkyo, Nagasaki, 852-8521, Japan

b School of Engineering, Nagasaki University 1-14 Bunkyo, Nagasaki, 852-8521, Japan

Corresponding Authors:

E-mail: h-tahara@nagasaki-u.ac.jp

Tel/Fax: +81-95-819-2675

E-mail: sagara@nagasaki-u.ac.jp

Tel: +81-95-819-2676, Fax: +81-95-819-2675

Table of Contents

S1. Experimental method

S2. DSC thermograms of $[C_4VC_7][TFSI]_2$, $[CzC_4ImC_1][TFSI]$, and CT-IL (Fig. S1).

S3. Absorption spectra of diluted solution of the RAILs (Fig. S2).

S4. Equilibrium constant of CT complex formation in CT-IL (Fig. S3 and Table S1).

S5. Fluorescence spectra of $[CzC_4ImC_1][TFSI]$ in neat and diluted solution (Fig. S4).

S6. Water contents and IR spectra of RAILs (Table S2 and Fig. S5).

S7. Arrehnius plot of the viscosities and ionic conductivities (Fig. S6).

S8. Walden plots of the RAILs (Fig. S7).

S9. CV multicycles of neat $[CzC_4ImC_1][TFSI]$ in the potential region B (Fig. S8).

S10. Ionic conductivity apparatus (Fig. S9, Table S3).

S11. Two-electrode cell for electrochemical measurements (Fig. S10).

S12. Fitting diagrams of experimental CVs by the simple Nernst model (Fig. S11).

S13. Fitting diagrams of experimental CVs by the two redox-site model (Fig. S12).

S14. Uncompensated resistance effects of CVs by DigiElch (Fig. S13).

S15. Uncompensated resistance effects of Nernst analysis by DigiElch (Figs. S14 and S15).

S16. Scan rate dependence of CVs in neat $[C_4VC_7][TFSI]_2$ and CT-IL by DigiElch (Fig. S16).

S17. DFT models of viologen and carbazole for evaluating Gibbs energy of the CT complex formation (Fig. S17).

S1. Experimental method

Materials and Methods.

Tetrabutylammonium bromide, 1-methyl imidazole, 1-bromobutane, 9*H*-carbazole, and 1,4-dibromobutane were purchased from Tokyo Chemical Industry Co., Inc. Potassium bis(trifluoromethanesulfonyl)imide was purchased from Kanto Chemical Co., Inc. 1-Methyl imidazole was distilled prior to use. The others were used as received. Solvents were purchased from Nakalai Tesque, Inc., unless indicated otherwise. Acetonitrile used in the synthesis was dried over molecular sieve 4A and then distilled under N_2 prior to use. Ethanol was of HPLC grade and used as received. Dichloromethane, ethyl acetate, and toluene were of extra pure grade and distilled prior to use. Water was purified through an Ultra-pure water system, Milli-Q Plus (Millipore Co.). Its resistivity was over 18 MΩ cm. All other chemicals were of reagent grade and used as received. 1-Butyl-1'-heptyl-4,4'-bipyridinium di(bis(trifluoromethanesulfonyl)imide), $[C_4VC_7][TFSI]_2$, was prepared according to the literatures.[45,S1]

Synthesis of RAILs.

Synthesis of 1-butyl-3-methylimidazolium bromide [BMIM][Br]. A solution of 1-methyl imidazole (5.27 g, 64.2 mmol) and 1-bromobutane (20.0 g, 164 mmol) in dry acetonitrile (50 mL) was refluxed overnight under N_2 and then evaporated *in vacuo*. The residue was added into ethyl acetate and sonicated with a bath-type sonicator for 30 min to obtain the bromide as a white solid (12.8 g, 91 %). ^{1}H NMR (300 MHz, DMSO-d_6, δ): 9.24 (s, 1H), 7.82 (s, 1H), 7.75 (s, 1H), 4.19 (m, 2H), 3.85 (s, 3H), 1.77 (m, 2H), 1.30 (m, 2H), 0.91 ppm (t, 3H).

Synthesis of 1-butyl-3-methylimidazolium bis(trifluoromethanesulfonyl)imide [BMIM][TFSI]. A solution of potassium bis(trifluoromethanesulfonyl)imide (23.3 g, 73.0 mmol) in water (30

mL) was added dropwise into a solution of [BMIM][Br] (12.8 g, 58.4 mmol) in water (50 mL). The reaction mixture was stirred overnight at ambient temperature. A white precipitate generated was extracted several times with dichloromethane. The organic layers were combined and then washed with water until no bromide was detected from the water layer by a silver nitrate test. The organic layer was dried over anhydrous $MgSO_4$ and then evaporated *in vacuo* to obtain [BMIM][TFSI] as a colorless viscous liquid (yield 22.8 g, 93.1 %). No UV-vis absorption of neat [BMIM][TFSI] was observed within 290-800 nm with 0.2 mm cell, showing no remarkable residual bromides in the [BMIM][TFSI]. X-ray fluorescence signal for elemental analysis of bromides was also below the lower detection limit.

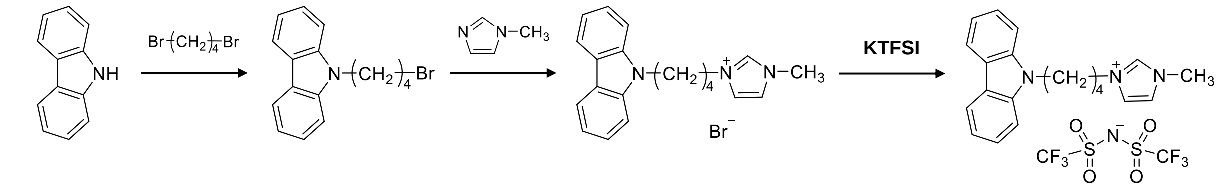

9*H*-carbazole **CzC_4Br** **[CzC_4ImC_1][Br]** **[CzC_4ImC_1][TFSI]**

Scheme S1. Synthesis scheme of the carbazole-based ionic liquid.

Synthesis of 9-(4-bromobutyl)carbazole (CzC_4Br). A solution of 9*H*-carbazole (2.21 g, 13.2 mmol), tetrabutylammonium bromide (0.427 g, 1.32 mmol), and 1,4-dibromobutane (15.0 mL, 125 mmol) in toluene (30 mL) was added into a 20 wt% KOH aqueous solution (30 mL). The mixture was vigorously stirred at 90 ^oC for 12 h. The organic layer was separated, washed with brine and then added dropwise into hexane to precipitate a white solid, which was recrystallized from ethanol to obtain CzC_4Br as a white needle crystal (yield 2.04 g, 50 %). ^1H NMR (300 MHz, $CDCl_3$, δ): 8.11 (m, 2H), 7.44 (m, 4H), 7.24 (m, 2H), 4.36 (t, 2H), 3.38 (t, 2H), 2.06 (m, 2H), 1.92 ppm (m, 2H).

Synthesis of 1-(4-(9-carbazolyl)butyl)-3-methylimidazolium bromide [CzC_4ImC_1][Br]. A solution of CzC_4Br (1.50 g, 4.56 mmol) and 1-methylimidazole (1.10 mL, 13.2 mmol) in dry acetonitrile (30 mL) was stirred overnight at 70 °C under N_2. After cooling down to ambient temperature, ethyl acetate was added into the reaction mixture to precipitate a white powder, which was filtered off and washed with ethyl acetate to obtain [CzC_4ImC_1][Br] as a white powder (yield 1.40 g, 70 %). mp. 141 °C (by DSC). ^1H NMR (300 MHz, $CDCl_3$, δ): 10.2 (s, 1H), 8.07 (d, 2H), 7.43 (m, 4H), 7.22 (t, 2H), 7.10 (s, 1H), 6.90 (s, 1H), 4.40 (t, 2H), 4.10 (t, 2H), 3.91 (s, 3H), 1.92 ppm (m, 4H).

Synthesis of *1-(4-(9-carbazolyl)butyl)-3-methylimidazolium bis(trifluoromethanesulfonyl)imide [CzC_4ImC_1][TFSI].* A solution of potassium bis(trifluoromethanesulfonyl)imide (2.93 g, 9.19 mmol) in water (7 mL) was added dropwise into a solution of [CzC_4ImC_1][Br] (1.20 g, 4.58 mmol) in water (50 mL). The reaction mixture was stirred overnight at ambient temperature. A white precipitate generated was extracted several times with dichloromethane. The organic layers were combined and then washed with water until no bromide was detected from water layer by a silver nitrate test. The organic layer was dried over anhydrous $MgSO_4$, filtered, and then evaporated *in vacuo* to obtain **[CzC_4ImC_1][TFSI]** as a colorless viscous liquid (yield 1.26 g, 73 %). T_g −23 °C. FAB-MS (*m/z*): Calcd. for $C_{22}H_{22}F_6N_4O_4S_2$, 584.5; found, 584.2. Anal. calcd. for $C_{22}H_{22}F_6N_4O_4S_2$: C, 45.20; H, 3.79; N, 9.59. Found: C, 45.35; H, 3.53; N, 9.55%. d_{25}, 1.6 g cm^{-3}. Conc.: Calcd. for $C_{22}H_{22}F_6N_4O_4S_2$ (**[CzC_4ImC_1][TFSI]**) 2.7 M.

Preparation of CT-IL. Equimolar amounts of **[CzC_4ImC_1][TFSI]** and **[C_4VC_7][TFSI]$_2$** were dissolved in acetonitrile, followed by stirring overnight at ambient temperature in the dark. After evaporation at 40 °C, a viscous brown liquid was obtained. T_g −34 °C. d_{25}, 1.3 g cm^{-3}. Conc.: Calcd. for $C_{22}H_{22}F_6N_4O_4S_2$ (**[CzC_4ImC_1][C_4VC_7][TFSI]$_3$**) 0.92 M.

^1H NMR for water content evaluations.

Water content in the ionic liquids (ILs) were evaluated by ^{1}H NMR as following procedures. The dried ILs (64.5 mg (0.0739 mmol) [C_4VC_7][TFSI]$_2$, 52.9 mg (0.0902 mmol) [CzC_4ImC_1][TFSI]$_2$, and 54.8 mg (0.0376 mmol) CT-IL) were dissolved in 0.75 mL DMSO-d_6 (from Sigma Aldrich, provided in ampoule, 99.96 atom% D, Lot. Number MKBK3605V) and then inserted into NMR tubes (Wilmad, for 500 MHz). ^{1}H NMR spectra were recorded by a JEOL JNM-ECZ400R NMR spectrometer with 90° pulse and 8 s pulse-duration at room temperature (22 °C). To evaluate intrinsic water content from sample manipulation, ^{1}H NMR of the blank (DMSO-d_6 only) was also measured. Signals of ^{1}H NMR were normalized by the integration of the peak at δ 2.50 assigned to the solvent residual peak to compare with each sample. Quantification of the water contents were evaluated by the integration of the peak at δ 3.33 assigned to water. The results were shown in Table S2.

IR measurement.

IR measurements were conducted with a JASCO IR-4600ST IR-spectrophotometer. The dried ionic liquids were dropped on an ATR unit (ATR Pro One with a diamond window) and the spectra were recorded with ATR-correction of the penetration depth and without H_2O correction. The IR spectra were shown in Figure S5.

Thermophysical measurements.

Melting point and glass transition point (T_g) of the compounds were determined by differential scanning calorimetry (DSC) with Shimadzu DSC-60 and a melting point apparatus (Yanaco MP-500P). T_g was determined by the inflection point of the first heating process. The results were shown in Fig. S1.

Sample preparation for the measurements.

$[C_4VC_7][TFSI]_2$, $[CzC_4ImC_1][TFSI]$, CT-IL, and the other solutions were dried *in vacuo* over 6 hours at 40 °C for $[CzC_4ImC_1][TFSI]$ and CT-IL, and 90 °C for $[C_4VC_7][TFSI]_2$ and the other ionic liquid solutions before the all measurements.

Density measurement.

The density of the ionic liquid was simply evaluated by the weight per unit volume, which was measured with a MICROMAN® M25 (Gilson) at room temperature (25 °C).

UV-vis and fluorescence measurements.

Transmission absorption measurements were conducted with a JASCO V-670 spectrophotometer. Fluorescence measurements were conducted with Shimadzu RF 5300-PC spectrofluorometer. A quartz cell with 1 cm path length was used for diluted solution. A quartz cell with 1 mm path length was used for evaluation of the absorption coefficient of CT complex between $[CzC_4ImC_1][TFSI]$ and $[C_4VC_7][TFSI]_2$. A quartz cell (Starna Scientific Ltd.) with 0.02 mm path length was used for the neat ionic liquid system. The thin optical path length was calibrated by the interference pattern of the transmission spectrum of the empty cell. Temperature of the samples for absorption measurements was controlled by electric Peltier unit apparatus.

Viscosity measurements.

Viscosity measurements of $[CzC_4ImC_1][TFSI]$ and CT-IL were conducted by a Brookfield corn plate viscometer (DV2T) with CPA-52Z and CPA-51Z spindles in a temporary glove box under N_2 to avoid moisture. The viscosities were determined within the Newtonian fluid region where the shear velocity is independent on the rotating speed. The temperature was controlled by a constant temperature water unit within 0.1 K variation (EYELA NCB-1200). The viscosity data of $[C_4VC_7][TFSI]_2$ was taken from our previous report.[45]

Ionic conductivity measurements.

Ionic conductivity measurements were performed by alternating current (AC) impedance technique with a potentiostat (HECS 9153, Huso, Japan) combined with a lock-in-amplifier (EG&G 7265) controlled by a personal computer with a home-made program written in Visual Basic for Application (VBA) language. The home-made cell (experimental cell constant 0.543 cm^{-1} calibrated by 0.01 mol kg^{-1} KCl aqueous solution at 298 K, see Fig. S8, and Table S2) was used for ionic conductivity measurement. The modulation amplitude was set to 0.1 Vrms and the conductivity was determined by extrapolation to real axis in Nyquist diagram. The temperature was controlled by a constant temperature water unit within 0.1 K variation. The conductivity data of $[C_4VC_7][TFSI]_2$ was reported in ref 45, but we herein showed the data of $[C_4VC_7][TFSI]_2$ using the above cell.

Computational calculations.

Standard DFT calculations were carried out using Gaussian 16[S2]. In general, geometries were optimized with the B3-LYP/6-31+G(d,p) procedure[S3], with the SMD-GIL solvation model[S4] included in these computations in conjunction with the [BMIM][TFSI] parameters. Optimized geometries of the model compounds were shown in Fig. S17. Following each geometry optimization, harmonic frequency analysis was carried out to confirm the nature of the stationary point as an equilibrium structure. To obtain zero-point vibrational energies and thermal corrections to 298 K Gibbs energies, we used unscaled B3-LYP frequencies. For the calculation of single-point energies, we used the more advanced MN15 functional[S5] in conjunction with the larger 6-311+G(3df,2p) basis set. Improved solvation effects were obtained at the M05-2X/6-31G(d) level[S6] using the SMD-GIL model and BMIM–TFSI parameters, which were then incorporated into the gas-phase MN15 energies. Computed relative energies (ΔG^{o}) in the text are given as MN15/6-311+G(3df,2p) 298 K Gibbs energies. Fragment molecular orbital (FMO)

analysis was performed to illuminate CT interaction between carbazole and viologen by AOMix software[S7,S8] without basis set superposition error correction. The analysis was conducted with M06-2X/6-311G(2d,p) level of theory in gas phase.

Electrochemical measurements.

Electrochemical measurements were performed by a potentiostat (HECS 9094, Huso) with a head box (HECS 972-1, Huso) for microelectrode experiments. Cyclic voltammetry was carried out with a home-made cell equipped with water jacket with two-electrodes configuration (Fig. S10). Electrode potential of working electrode was controlled against a counter electrode. This two-electrodes configuration was applicable for very small working electrode with very large counter electrode which can compensate a current of the working electrode with reducing polarization of the counter electrode potential.[S9] An Au microelectrode (d = 10 μm, BAS Inc.) as working electrode were polished with 0.05 μm alumina suspension on a buff and lightly wiped with a wipe, and then the electrodes were washed with water and then rinsed with acetonitrile. Pt plate as a quasi-reference and counter electrode were used after flame-annealing and cooled down under Ar (99.998% purity) atmosphere. Two-electrodes configuration were illustrated in Fig. S9. The sample solution was dropped on the Pt plate and then contacted with Au microelectrode in manner of hanging meniscus configuration.

Electrochemical reversibility.

Here, for readers' better understanding, we added comments on two experimental conditions regarding cyclic voltammograms (CVs).

First, we used Au microelectrode to reduce iR drop from huge resistance of our RAILs. The static solution resistance (R_s) of the solution for a disk electrode with radius (r) is expressed by the conductivity (σ).[S10]

$$R_s = \frac{1}{4r\sigma} \tag{13}$$

For example, at 333 K with $r = 5.0\times10^{-4}$ cm, R_s is 0.62 MΩ for $[C_4VC_7][TFSI]_2$ ($\sigma = 8.0\times10^{-4}$ S cm^{-1}) and 1.1 MΩ for CT-IL ($\sigma = 4.5\times10^{-4}$ S cm^{-1}). Possible iR drop of each system can be negligible for analysis of electrochemical reversibility, because current amplitudes were in this study at most around 10 nA, i.e. around 1 mV iR dorp (see Figs. S14 and S15).

Second, CVs in normal scan rate did not exhibit sigmoidal but peaked waveform, because the RAILs are very viscous and the (apparent) diffusion coefficients are quite low causing formation of not a spherical (radial) but a linear (planar) diffusion layer. Dimensionless sphericity parameter (σ_s) of diffusion layer is described as following equation.[S11,S12]

$$\sigma_s = \sqrt{\frac{RTD}{nFvr^2}} \tag{14}$$

where, variables are the conventional meanings, particularly D and v are diffusion coefficient and scan rate, respectively. Planar diffusion ($\sigma_s < 0.012$) and radial diffusion ($\sigma_s > 40$) are numerically parameterized, whereas the mixed diffusion can be depicted between them. For example, the diffusion coefficients of $[C_4VC_7][TFSI]_2$ and CT-IL were evaluated by the steady state CVs of Fig. 5 and Table 2 to be respectively 2.4×10^{-8} and 1.0×10^{-8} cm^2 s^{-1}, resulting in the following values. In the case of $[C_4VC_7][TFSI]_2$ at 333 K, σ_s values are 0.23 at 50 mV s^{-1} and 5.2 at 0.1 mV s^{-1}. In the case of CT-IL at 333 K, σ_s are 0.15 at 50 mV s^{-1} and 1.5 at 0.5 mV s^{-1}. Note that the above values were evaluated with the apparent diffusion coefficients without migration correction due to absence of the supporting electrolyte.[21] Although the sphericity parameters were still within the mixed diffusion region at the slow scan rate, the CVs can be approximately regarded as Nernstian sigmoid waveform (Fig. S16).

S2. DSC thermograms of [C_4VC_7][TFSI]$_2$, [CzC_4ImC_1][TFSI], and CT-IL.

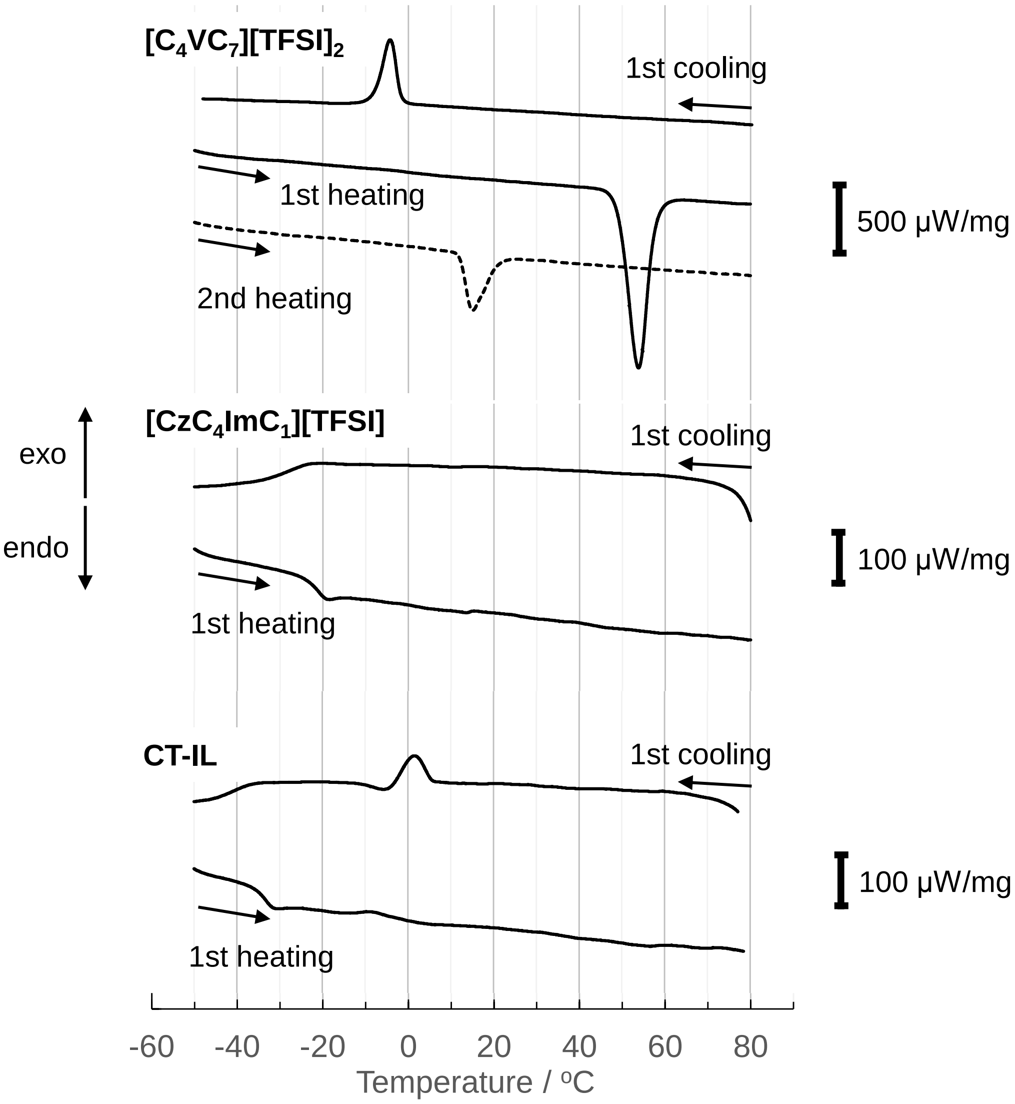

Fig. S1. DSC thermograms of [C_4VC_7][TFSI]$_2$, [CzC_4ImC_1][TFSI], and CT-IL at 10 °C min^{-1}.

S3. Absorption spectra of diluted solution of the RAILs.

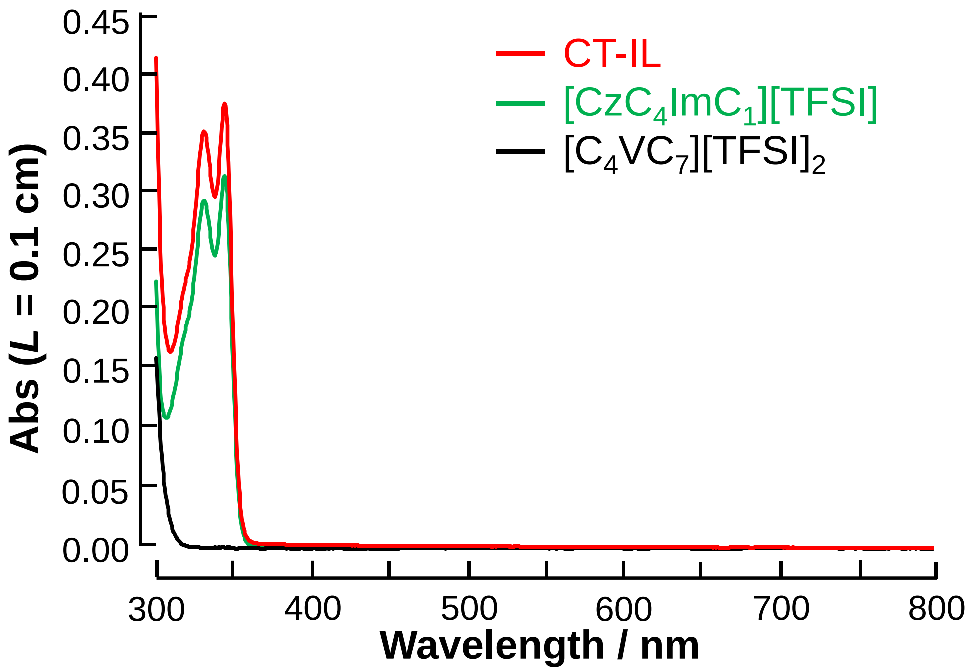

Fig. S2. Absorption spectra of $[CzC_4ImC_1][TFSI]$, $[C_4VC_7][TFSI]_2$, and CT-IL diluted solution in acetonitrile (0.1 cm optical path length, 1 mM concentration) at room temperature (297 K).

S4. Equilibrium constant of CT complex formation in CT-IL.

The thermodynamic parameters of the CT complex were estimated by following equations.

$$\mathrm{Abs}_{\mathrm{CT}} = \varepsilon_{427\mathrm{nm}} L c_{\mathrm{CT}} \tag{S1}$$

$$\mathrm{Cz} + \mathrm{V}^{++} \rightleftharpoons \mathrm{CzV}^{++}, \quad K_{\mathrm{CT}} = \frac{c_{\mathrm{CT}}}{(c_0 - c_{\mathrm{CT}})^2} \tag{S2}$$

$$\frac{\Delta G}{T} = -R \ln K_{\mathrm{CT}} \tag{S3}$$

$$\Delta H = \left(\frac{\partial(\Delta G/T)}{\partial(1/T)} \right)_p \tag{S4}$$

$$\Delta G = \Delta H - T\Delta S \tag{S5}$$

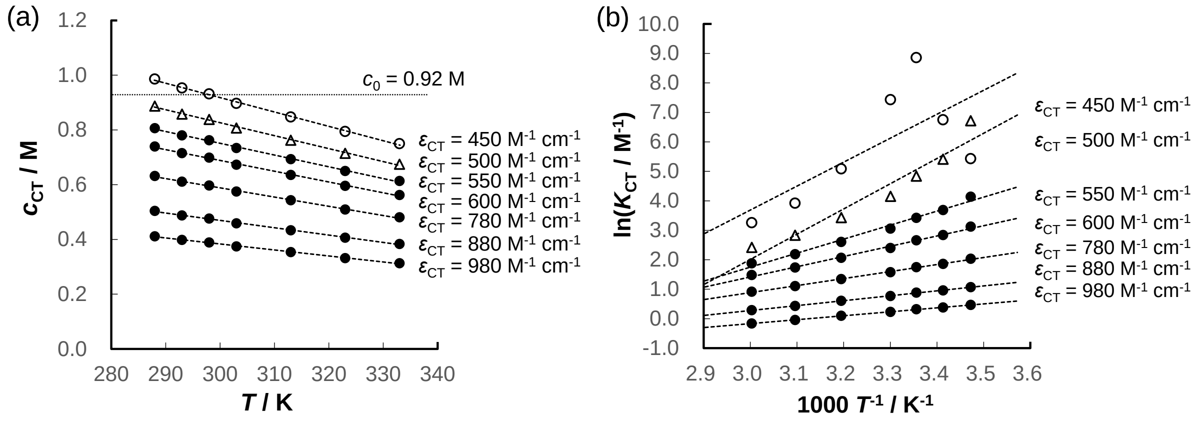

Fig. S3. (a) Temperature dependent concentration of the CT complex on assumption of several molar absorption coefficients at 427 nm ($\varepsilon_{\mathrm{CT}}$) using L = 20 μm. (b) Arrhenius plot of K_{CT} evaluated by temperature dependent absorption spectra with assumption of several molar absorption coefficients of the CT transition.

Table S1. The evaluated thermodynamic parameters of CT-IL in Figs. S2 at 298 K on the assumption of the molar absorption coefficient (ε_{CT}) at 427 nm. Note that the analytical concentration (c_0) is 0.92 M in CT-IL.

ε_{CT} / M^{-1} cm^{-1}	c_{CT} / M	ΔH / kJ mol^{-1}	ΔS / J mol^{-1} K^{-1}	ΔG(298 K) / kJ mol^{-1}	K_{CT} / M^{-1}	R^2
450	0.932	-67.6	172	-21.9	7000	0.4946
500	0.838	-71.2	197	-12.0	126	0.9348
550	0.762	-39.4	104	-8.5	31	0.9862
570	0.735	-34.2	89.2	-7.6	22	0.9907
600	0.699	-28.9	74.8	-6.6	14	0.9942
**780**	**0.537**	**-19.8**	**52.0**	**-4.3**	**5.7**	**0.9988**
**880**	**0.476**	**-13.9**	**39.4**	**-2.2**	**2.4**	**0.9991**
**980**	**0.428**	**-11.1**	**34.8**	**-0.79**	**1.4**	**0.9992**
1200	0.349	-10.1	33.6	-0.17	1.1	0.9991
1400	0.299	-9.1	32.6	0.62	0.78	0.9990
(DFT)		-49.8	155	-3.6	4.3	

S5. Fluorescence spectra of [CzC_4ImC_1][TFSI] in neat and diluted solution.

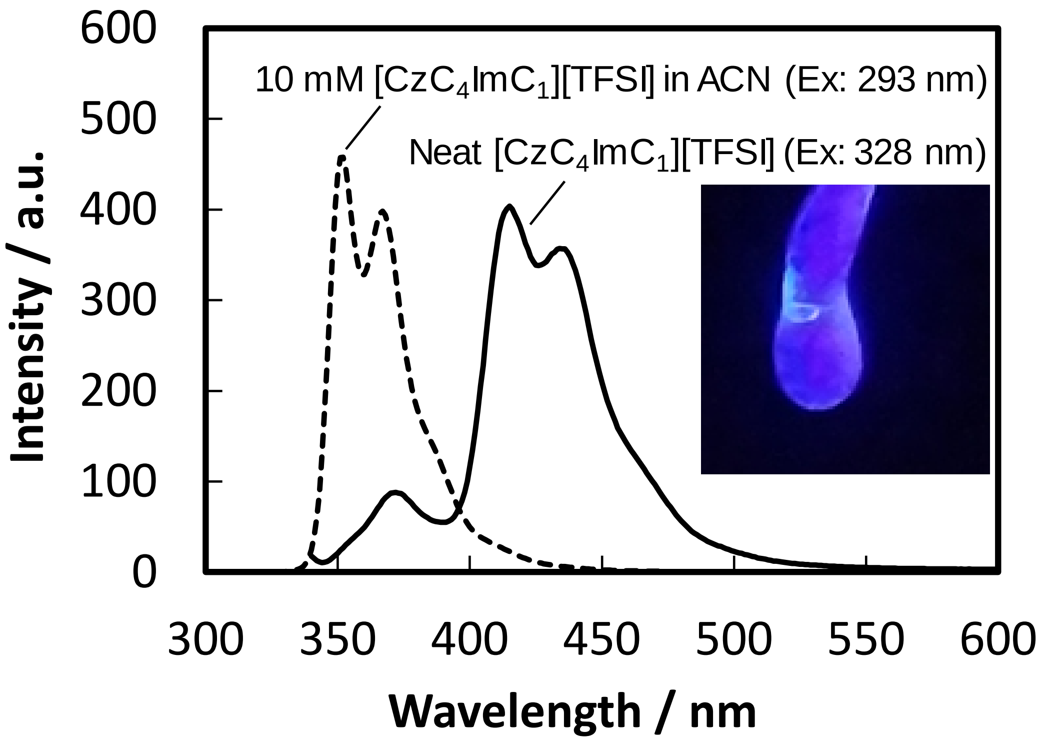

Fig. S4. Fluorescence spectra of neat [CzC_4ImC_1][TFSI] (excitation wavelength 328 nm, solid line) and 10 μM [CzC_4ImC_1][TFSI] in acetonitrile (excitation wavelength 293 nm, dash line) at room temperature. The inset picture is of the neat [CzC_4ImC_1][TFSI] irradiated by a UV light (365 nm).

S6. Water contents and IR spectra of RAILs.

Table S2. Water contents (weight concentration) in the neat RAILs evaluated by ^{1}H NMR

$[C_4VC_7][TFSI]_2$	$[CzC_4ImC_1][TFSI]$	CT-IL
0.098 wt%	0.55 wt%	0.27 wt%

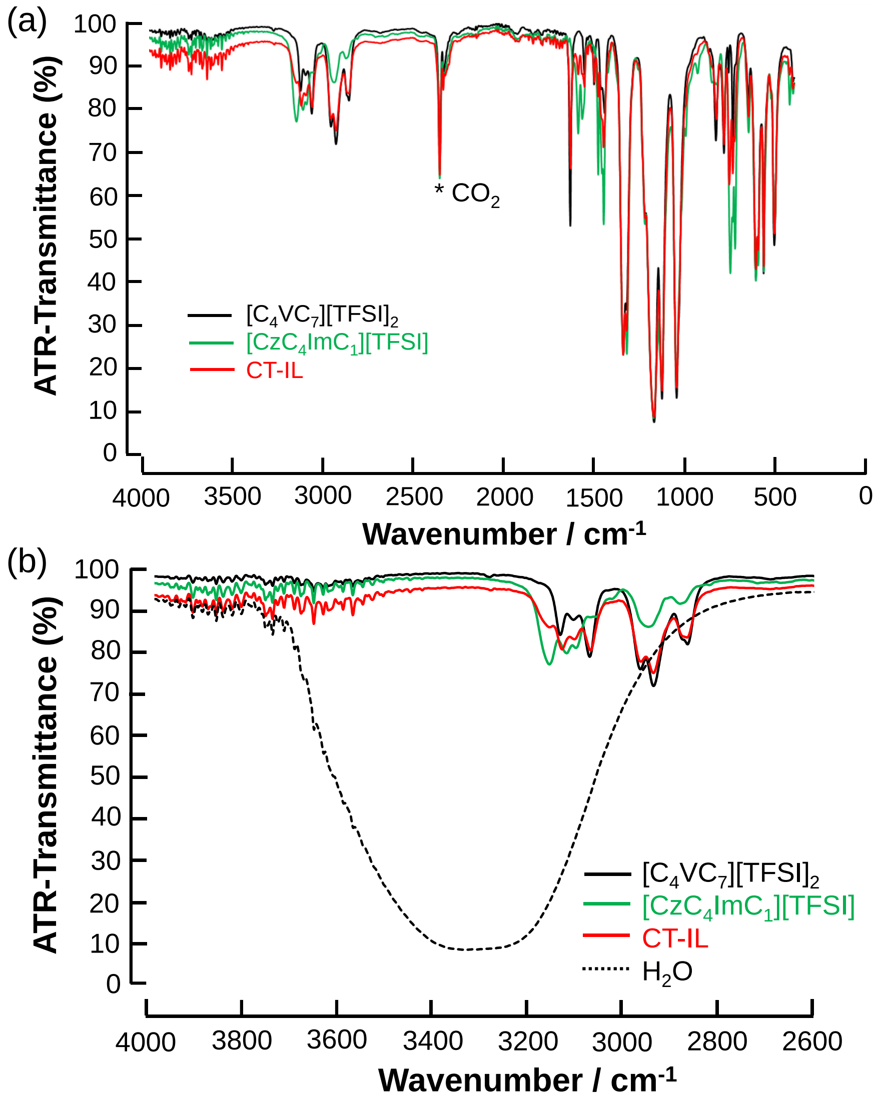

Fig. S5. ATR-IR spectra of the neat $[C_4VC_7][TFSI]_2$, $[CzC_4ImC_1][TFSI]$, and CT-IL. (a) the whole region, (b) the expanded region of water. The ATR-IR spectrum of pure water was displayed in (b) for comparison.

S7. Arrhenius plot of viscosity and ionic conductivity.

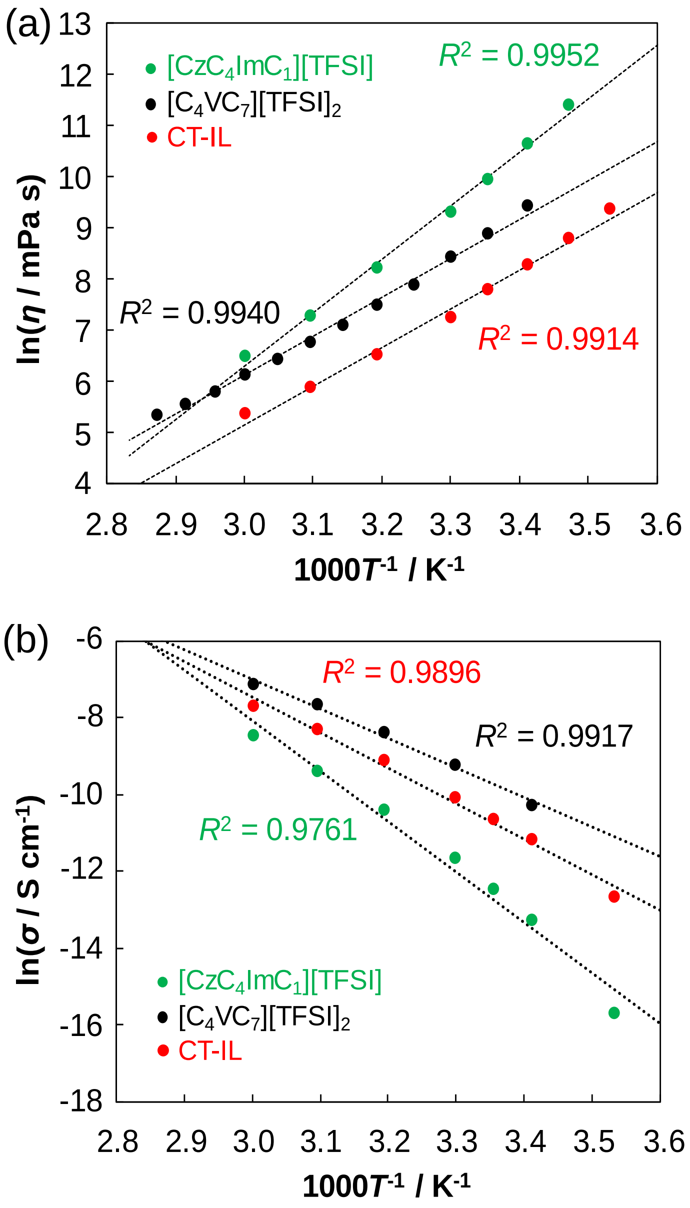

Fig. S6. Arrhenius plots of (a) viscosity and (b) ionic conductivity of neat [CzC$_4$ImC$_1$][TFSI], neat [C$_4$VC$_7$][TFSI]$_2$, and CT-IL.

S8. Walden plot of the RAILs.

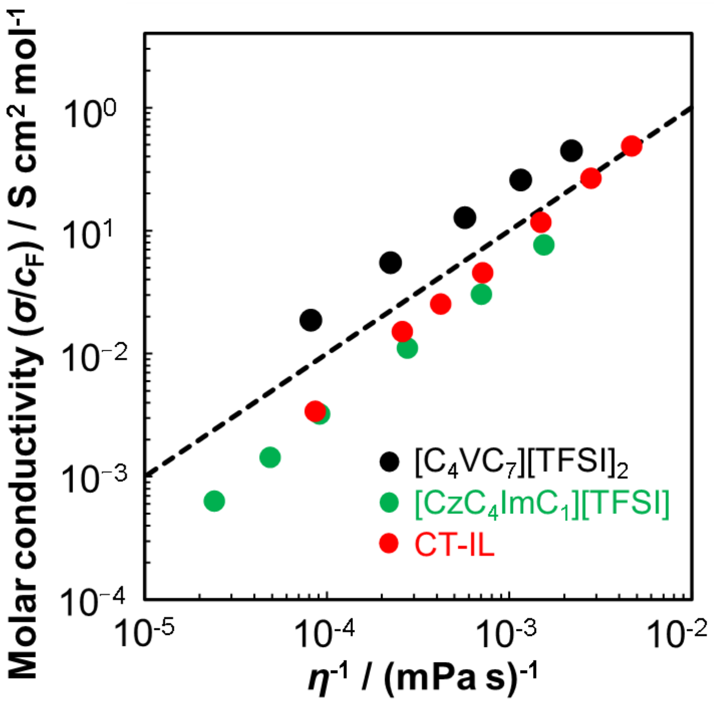

Fig. S7. Walden plot of the RAILs. The dashed line is the ideal Walden line.

S9. CV multicycles of neat [CzC$_4$ImC$_1$][TFSI] in the potential region B.

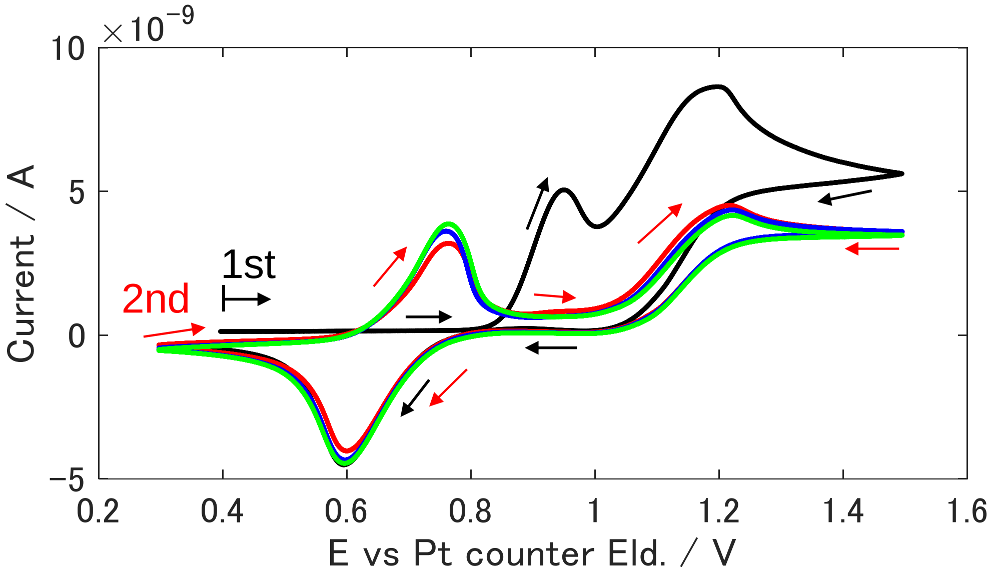

Fig. S8. CV multicycles of neat [CzC$_4$ImC$_1$][TFSI] in the potential region B (redox of carbazole and the electro-polymerization) at 333 K and 50 mV s^{-1}. The color indicates the cycle number, black for the first scan, red for the second scan, blue for the third scan, and green for the fourth scan.

S10. Ionic conductivity apparatus.

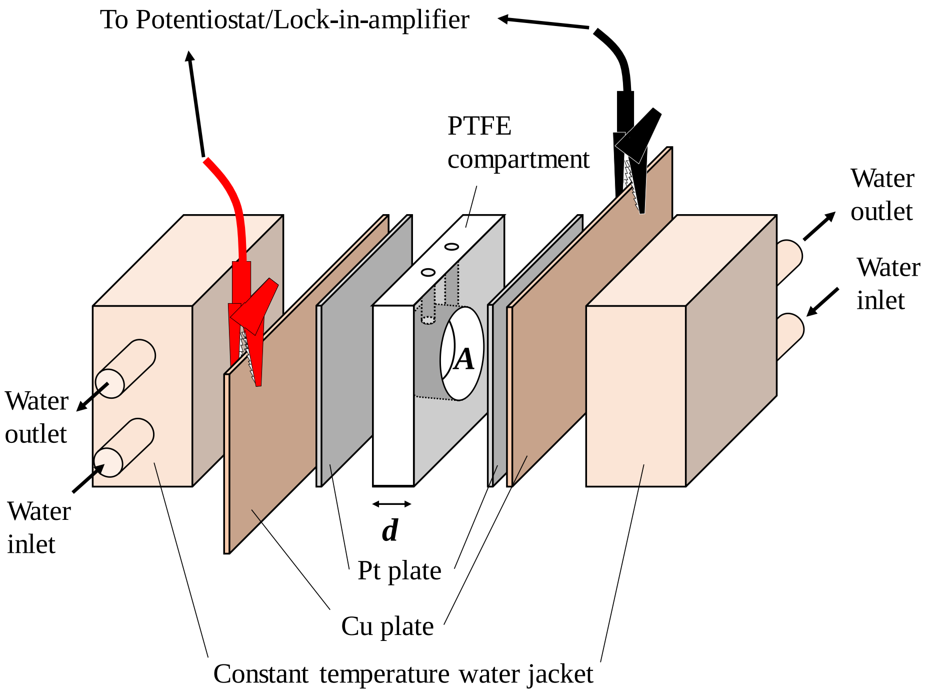

Fig. S9. Home-made cell for ionic conductivity measurement.

Table S3. Specification of the home-made cell for ionic conductivity. The experimental cell constant (K_{exp}) was determined by 0.01 mol kg^{-1} KCl aqueous solution ($\sigma = 0.140$ S m^{-1}) at 298K.

Thickness d / cm	Diameter / cm<br>Area A / cm^2	Theor. Cell Constant<br>K (= d / A) / cm^{-1}	Exp. Cell Constant<br>K_{exp} / cm^{-1}
0.416	0.990<br>0.770	0.540	0.543

S11. Two-electrode cell for electrochemical measurements.

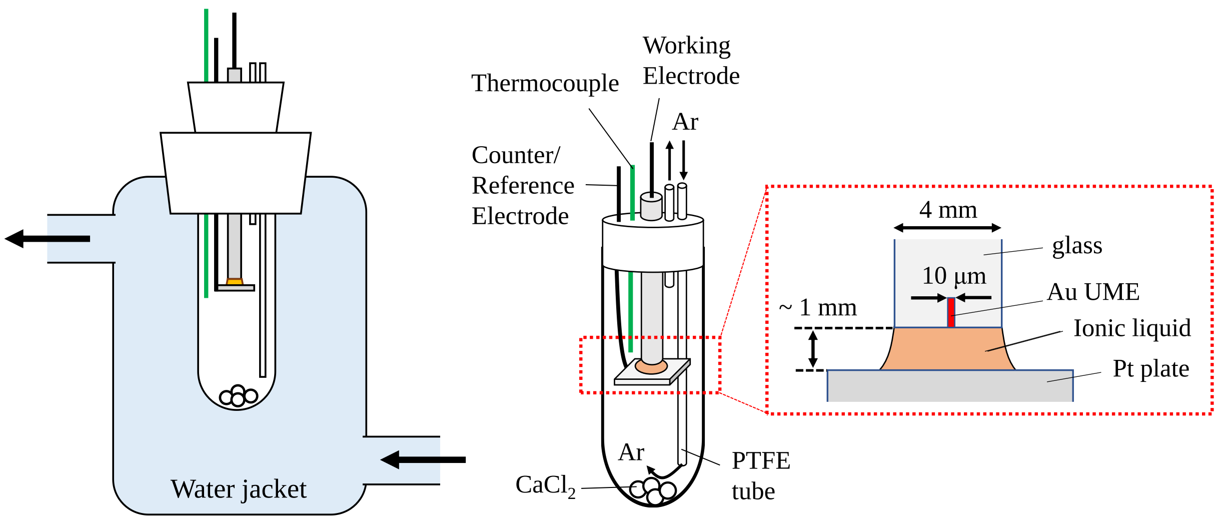

Fig. S10. Home-made two-electrode cell for voltammetry under constant temperature.

S12. Fitting diagrams of experimental CVs by the simple Nernst model.

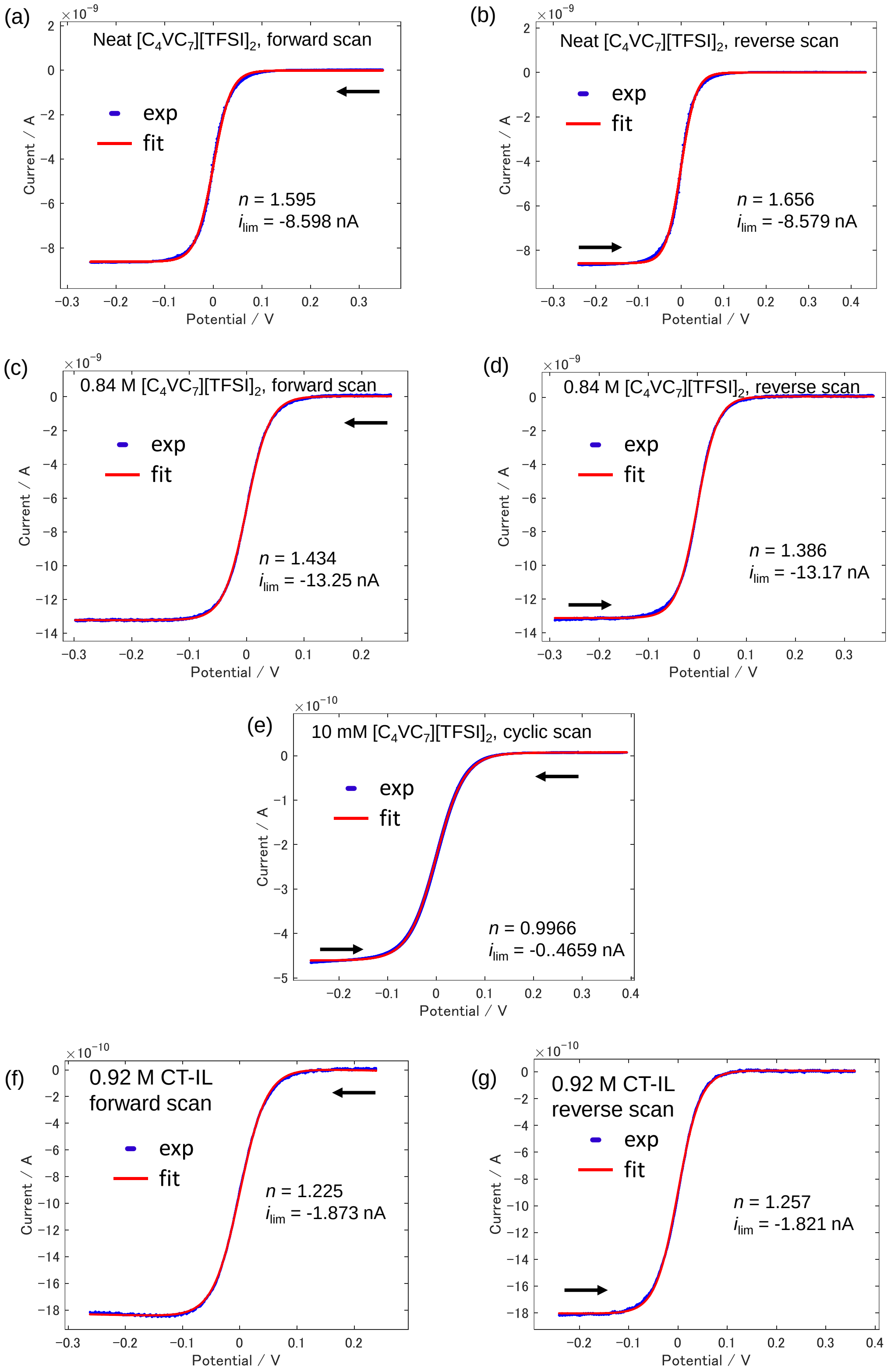

Fig. S11. Fitting diagrams of CVs at 60 °C by simple Nernstian model in various $[C_4VC_7][TFSI]_2$ systems.

S13. Fitting diagrams of experimental CVs by the two redox-site model.

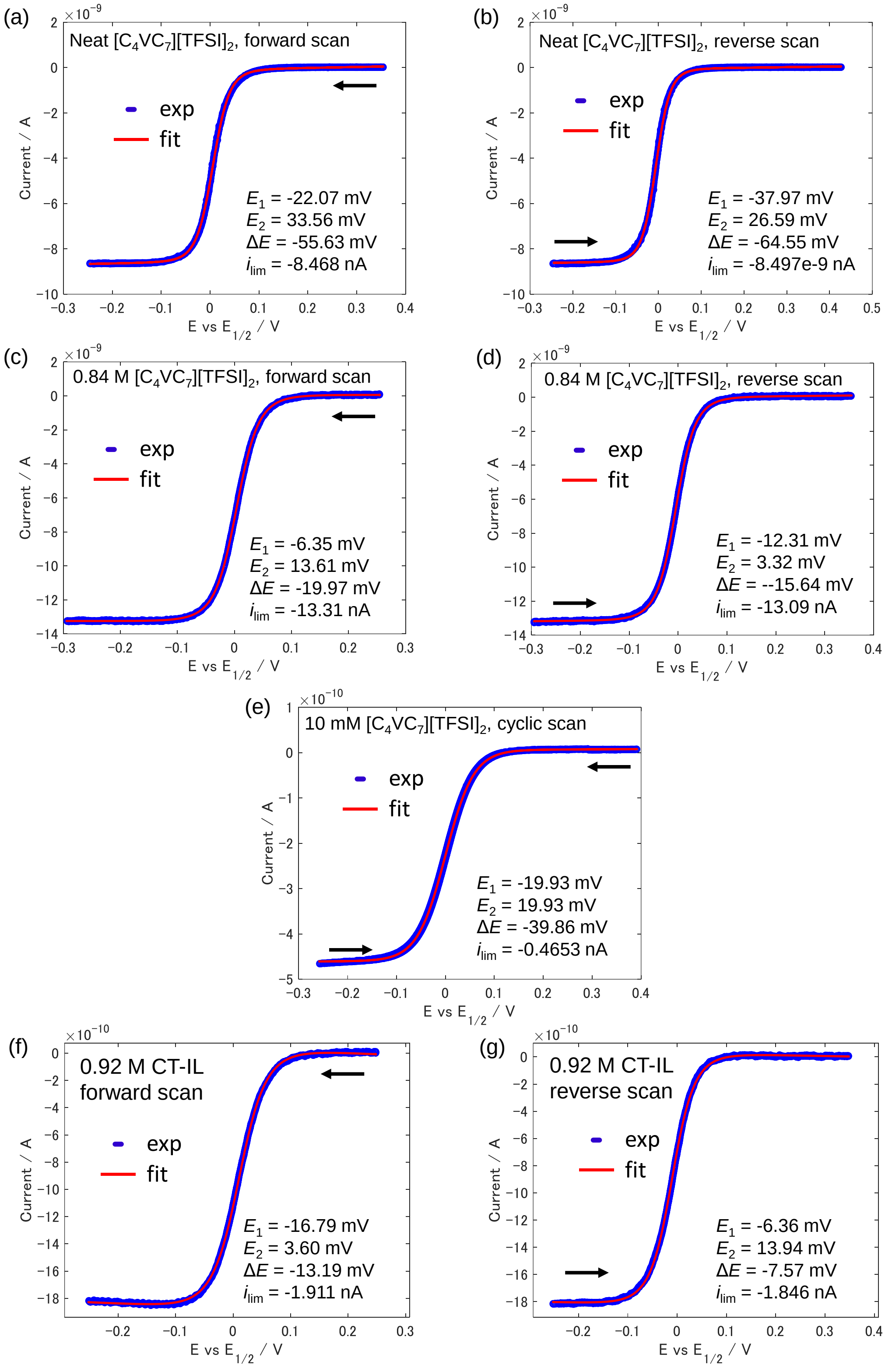

Fig. S12. Fitting diagrams of CVs at 60 ºC by the two redox-site model in various $[C_4VC_7][TFSI]_2$ systems.

S14. Uncompensated resistance effects of CVs by DigiElch.

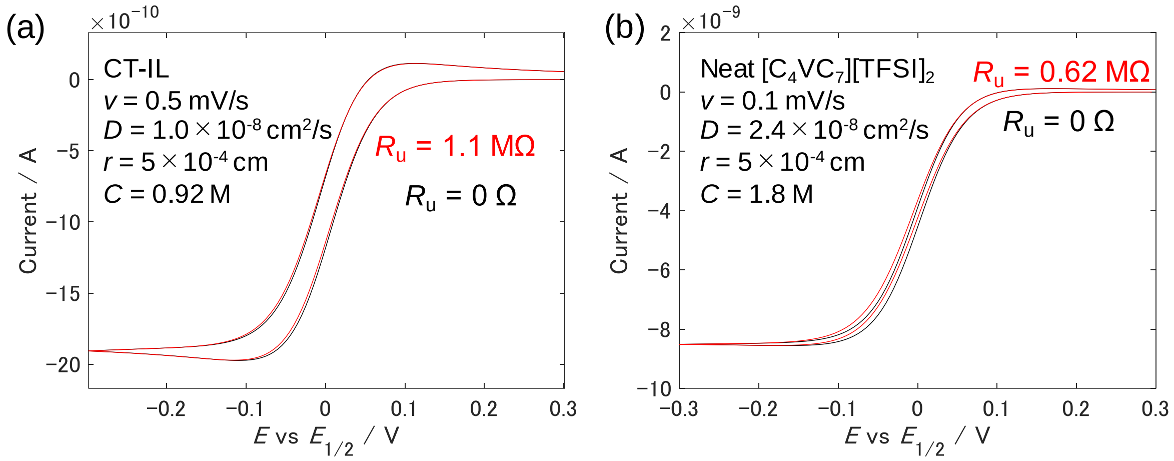

Fig. S13. Simulated CVs of CT-IL and neat $[C_4VC_7][TFSI]_2$ with and without uncompensated solution resistance (R_u) by DigiElch Professional software. The parameters are set to $k^0 = 1{\times}10^5$ cm/s, α = 0.5, T = 333 K, and semi-infinite 2D diffusion model with disk geometry. No assumption was considered as CT complex and dimerization.

S15. Uncompensated resistance effects of Nernst analysis by DigiElch.

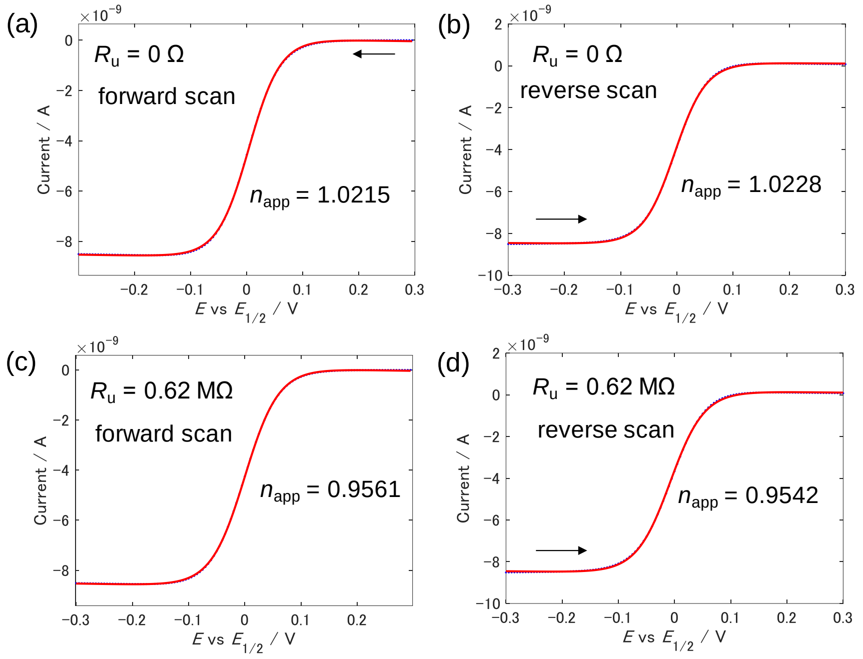

Fig. S14. Simulated CVs (blue dot) and their Nernst analysis (red line) of neat $[C_4VC_7][TFSI]_2$ with and without uncompensated solution resistance (R_u) by DigiElch Professional software. The parameters are set to $k^0 = 1\times10^5$ cm/s, $\alpha = 0.5$, $T = 333$ K, and semi-infinite 2D diffusion model with disk geometry. No assumption was considered as CT complex and dimerization.

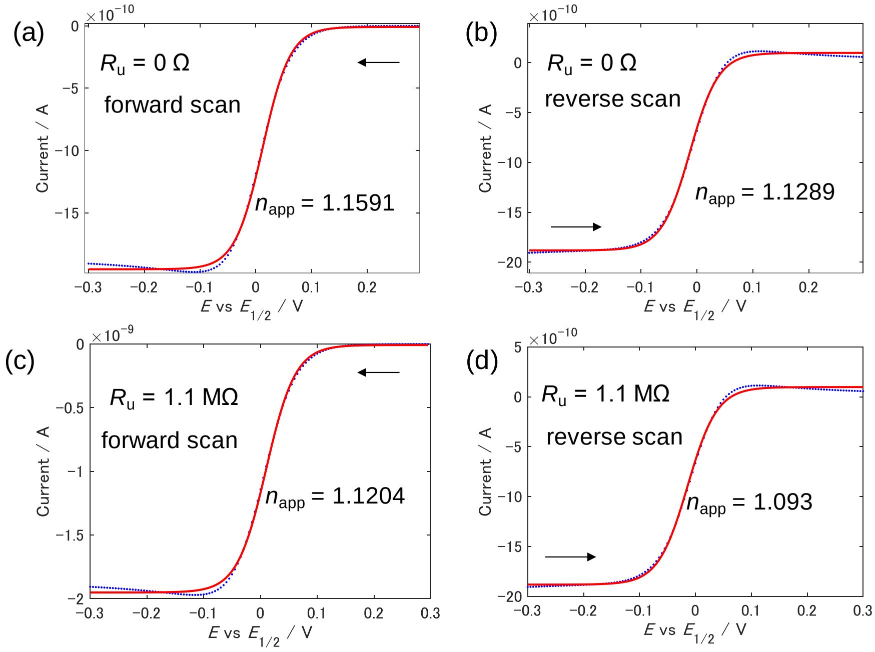

Fig. S15. Simulated CVs (blue dot) and their Nernst analysis (red line) of CT-IL with and without uncompensated solution resistance (R_u) by DigiElch Professional software. The parameters are set to $k^0 = 1\times10^5$ cm/s, $\alpha = 0.5$, $T = 333$ K, and semi-infinite 2D diffusion model with disk geometry. No assumption was considered as CT complex and dimerization.

S15. Scan rate dependence of CVs in neat $[C_4VC_7][TFSI]_2$ and CT-IL by DigiElch.

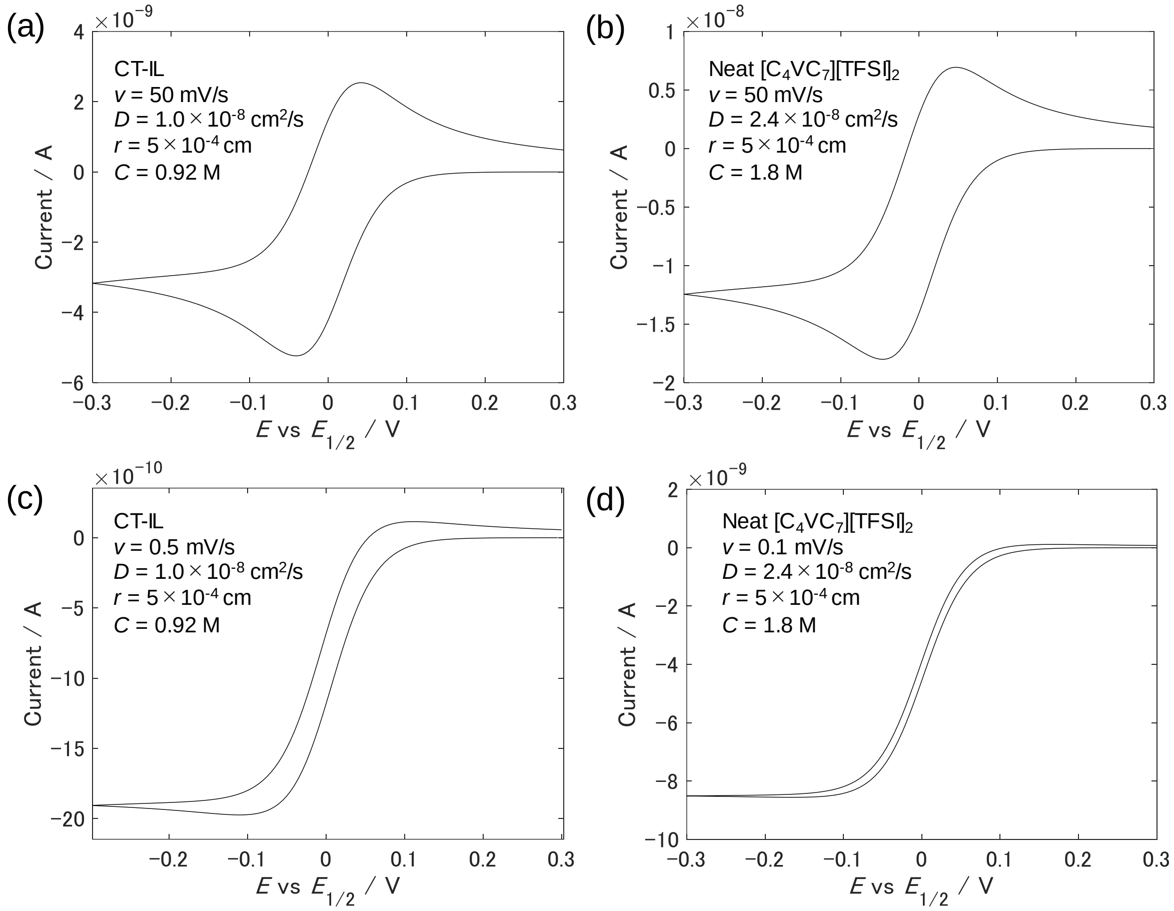

Fig. S16. Simulated CVs of neat $[C_4VC_7][TFSI]_2$ and CT-IL. The parameters are set to $k^0 = 1\times10^5$ cm/s, $\alpha = 0.5$, $T = 333$ K, and semi-infinite 2D diffusion model with disk geometry by DigiElch Professional software. Dimerization, CT complex formation, and *iR* compensation were not assumed.

S17. DFT models of a viologen, carbazole, and TFSI for evaluating intermolecular interactions

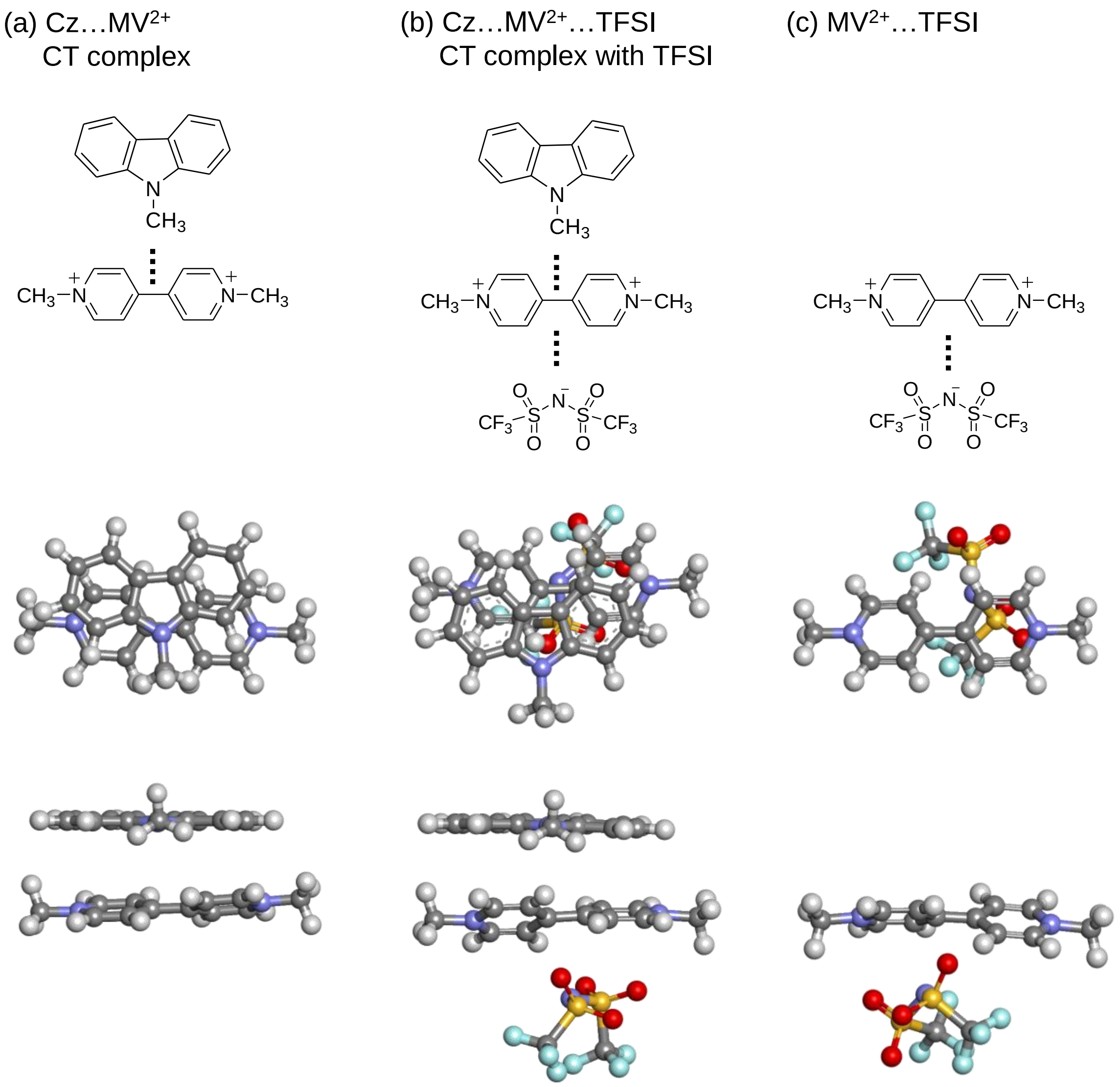

Fig. S17. DFT optimized structures of the model compounds of the complexes. (a) the CT complex between dimethylviologen and 9-methyl carbazole, (b) the CT complex attracted with TFSI anion, and (c) dimethylviologen attracted with TFSI anion. The calculation details were described in S1 Experimental method section.

References

(S1) H. Tahara, Y. Furue, C. Suenaga, T. Sagara, *Cryst. Growth Des.*, 2015, **15**, 4735–4740.

(S2) Gaussian 16, Revision C.01, M. J. Frisch, G. W. Trucks, H. B. Schlegel, G. E. Scuseria, M. A. Robb, J. R. Cheeseman, G. Scalmani, V. Barone, G. A. Petersson, H. Nakatsuji, X. Li, M. Caricato, A. V. Marenich, J. Bloino, B. G. Janesko, R. Gomperts, B. Mennucci, H. P. Hratchian, J. V. Ortiz, A. F. Izmaylov, J. L. Sonnenberg, D. Williams-Young, F. Ding, F. Lipparini, F. Egidi, J. Goings, B. Peng, A. Petrone, T. Henderson, D. Ranasinghe, V. G. Zakrzewski, J. Gao, N. Rega, G. Zheng, W. Liang, M. Hada, M. Ehara, K. Toyota, R. Fukuda, J. Hasegawa, M. Ishida, T. Nakajima, Y. Honda, O. Kitao, H. Nakai, T. Vreven, K. Throssell, J. A. Montgomery, Jr., J. E. Peralta, F. Ogliaro, M. J. Bearpark, J. J. Heyd, E. N. Brothers, K. N. Kudin, V. N. Staroverov, T. A. Keith, R. Kobayashi, J. Normand, K. Raghavachari, A. P. Rendell, J. C. Burant, S. S. Iyengar, J. Tomasi, M. Cossi, J. M. Millam, M. Klene, C. Adamo, R. Cammi, J. W. Ochterski, R. L. Martin, K. Morokuma, O. Farkas, J. B. Foresman, and D. J. Fox, Gaussian, Inc., Wallingford CT, 2016.

(S3) P. J. Stephens, F. J. Devlin, C. F. Chabalowski, M. J. Frisch, *J. Phys. Chem.*, 1994, **98**, 11623–11627.

(S4) V. S. Bernales, A. V. Marenich, R. Contreras, C. J. Cramer, D. G. Truhlar, *J. Phys. Chem. B*, 2012, **116**, 9122–9129.

(S5) H. S. Yu, X. He, S. L. Li, D. G. Truhlar, *Chem. Sci.*, 2016, **7**, 5032–5051.

(S6) Y. Zhao, N. E. Schultz, D. G. Truhlar, *J. Chem. Theory Comput.*, 2006, **2**, 364–382.

(S7) The web site of AOMix software: http://www.sg-chem.net/aomix-s/

(S8) S. I. Gorelsky and E. I. Solomon, Theor. Chem. Acc., 2008, **119**, 57–65.

(S9) A. M. Bond, P. A. Lay, *J. Electroanal. Chem.*, 1986, **199**, 285–295.

(S10) J. Newman, *J. Electrochem. Soc.*, 1966, **113**, 501–502.

(S11) C. L. Bentley, A. M. Bond, A. F. Hollenkamp, P. J. Mahon, J. Zhang, *Anal. Chem.*, 2014, **86**, 2073–2081.

(S12) P. J. Mahon, W. R. C. Phillips, *Electrochim. Acta.*, 2012, **74**, 16–22.
